# CELF4 Regulates Translation and Local Abundance of a Vast Set of mRNAs, Including Genes Associated with Regulation of Synaptic Function

**DOI:** 10.1371/journal.pgen.1003067

**Published:** 2012-11-29

**Authors:** Jacy L. Wagnon, Michael Briese, Wenzhi Sun, Connie L. Mahaffey, Tomaž Curk, Gregor Rot, Jernej Ule, Wayne N. Frankel

**Affiliations:** 1 The Jackson Laboratory, Bar Harbor, Maine, United States of America; 2 MRC Laboratory of Molecular Biology, Cambridge, United Kingdom; 3 Faculty of Computer and Information Science, University of Ljubljana, Ljubljana, Slovenia; Stanford University School of Medicine, United States of America

## Abstract

RNA–binding proteins have emerged as causal agents of complex neurological diseases. Mice deficient for neuronal RNA–binding protein CELF4 have a complex neurological disorder with epilepsy as a prominent feature. Human *CELF4* has recently been associated with clinical features similar to those seen in mutant mice. CELF4 is expressed primarily in excitatory neurons, including large pyramidal cells of the cerebral cortex and hippocampus, and it regulates excitatory but not inhibitory neurotransmission. We examined mechanisms underlying neuronal hyperexcitability in *Celf4* mutants by identifying CELF4 target mRNAs and assessing their fate in the absence of CELF4 in view of their known functions. CELF4 binds to at least 15%–20% of the transcriptome, with striking specificity for the mRNA 3′ untranslated region. CELF4 mRNA targets encode a variety of proteins, many of which are well established in neuron development and function. While the overall abundance of these mRNA targets is often dysregulated in *Celf4* deficient mice, the actual expression changes are modest at the steady-state level. In contrast, by examining the transcriptome of polysome fractions and the mRNA distribution along the neuronal cell body-neuropil axis, we found that CELF4 is critical for maintaining mRNA stability and availability for translation. Among biological processes associated with CELF4 targets that accumulate in neuropil of mutants, regulation of synaptic plasticity and transmission are the most prominent. Together with a related study of the impact of CELF4 loss on sodium channel Na_v_1.6 function, we suggest that CELF4 deficiency leads to abnormal neuronal function by combining a specific effect on neuronal excitation with a general impairment of synaptic transmission. These results also expand our understanding of the vital roles RNA–binding proteins play in regulating and shaping the activity of neural circuits.

## Author Summary

Epilepsy is a devastating brain disorder whereby a loss of regulation of electrochemical signals between neurons causes too much excitation and ultimately results in an “electrical storm” known as a seizure. Epilepsy can be heritable, but it is usually genetically complex, resulting from a collaboration of many genes. It is also a frequent feature of other common brain diseases, such as autism spectrum disorder and intellectual disability, likely because these diseases have a similar dysregulation of neuronal communication. To understand more about how the brain regulates electrical activity, we focused on an RNA–binding protein called CELF4, because a) mice that lack CELF4 have a complex form of epilepsy that includes features of other neurological diseases and b) this kind of protein has the potential to be a master regulator. We show that CELF4 binds to a vast array of mRNAs, and without CELF4 these mRNAs accumulate in the wrong places and can produce the wrong amount of protein. Moreover, many of these mRNAs encode key players in electrochemical signaling between neurons. Although the defects in individual mRNAs are modest, like a genetically complex disease, together these alterations collude to cause neurological symptoms including recurrent seizures.

## Introduction

Idiopathic epilepsies (IE) have an unknown etiology, but are generally accepted to be genetic in origin. Although ion channel defects have been identified as primary causal agents in some rare monogenic forms of IE, the genetic underpinnings of the vast majority of more common IE cases, which are likely to be genetically complex, remain unknown. Increasingly, molecular genetic studies have identified defects in non-ion channel genes in common IE, such as the calcium sensors *EFHC1* in juvenile myoclonic epilepsy (JME) and *CASR* in idiopathic generalized epilepsy (IGE) [1], [2], [3], [4]. Regulators of transcription, such as *BRD2* and *ELP4*, have been linked with JME and Rolandic epilepsy (RE), respectively [5], [6]. *LGI1*, a neuronal secreted protein, has been associated with epilepsy and schizophrenia [7], [8], [9]. Importantly for our study, epilepsy has a high rate of comorbidity with other complex neurodevelopmental disorders, such as autism spectrum disorders (ASD), intellectual disability (ID) and schizophrenia, suggesting that these disorders may indeed share many susceptibility genes [10], [11], [12], [13].

RNA-binding proteins (RBPs) are increasingly being associated with complex genetic diseases, as they can regulate the expression of many genes co-transcriptionally or post-transcriptionally via interactions with mRNA [14], [15], [16]. One such RBP is fragile X mental retardation protein (FMRP). Loss of FMRP causes fragile X syndrome (FXS), a disorder with symptoms including intellectual disability, features of autism, attention deficit and hyperactivity, and altered neuronal excitability that leads to seizures [17]. Additional mammalian RBPs associated with epilepsy in humans and/or mice include JRK/JH8, RBFOX1, PUMILIO-2, and CELF4 [18], [19], [20], [21], [22], [23], [24], [25]. CELF4 deficiency causes a range of neurological abnormalities in mice, including seizures, due to increased neuronal excitation [24], [25]. In human patients, *CELF4* is implicated in del(18q) syndrome phenotypes (discussed in [26]) including at least one patient with seizures, hyperactivity, and signs of autistic behaviors, carrying a translocation within *CELF4* itself [26].

CELF4 (CUGBP, ELAV-like family member 4) is one of six mammalian CELF proteins that function in mRNA metabolism. CELF1 and CELF2 are broadly expressed, while CELF3 and CELF5 are expressed primarily in the nervous system and CELF6 is expressed in brain, kidney, and testis [27], [28], [29]. Previously, CELF4 was reported to be expressed in many tissues [28], [29] however many studies including our initial description of CELF4 (then BRUNOL4) deficient mice indicate that CELF4 expression is restricted to the central nervous system across species, including mouse [25], [30], nematode (*Caenorhabditis elegans*) [31], chicken [32], and frog (*Xenopus laevis*) [33]. CELF proteins play various roles in co-transcriptional and post-transcriptional RNA processing [34]. All CELF proteins can affect pre-mRNA splicing, at least in cell-free assays, but individual CELFs have shown divergent roles in regulating mRNA stability and translation. CELF1 regulates mRNA stability by promoting deadenylation and degradation [35], [36], [37]. In contrast, CELF2 has been shown to bind the 3′ UTR of Cyclooxygenase-2 and Mcl-1 transcripts, stabilizing them and repressing their translation [38], [39]. The *Xenopus* orthologue of CELF3, however, binds the 3′ UTR of Cyclin A transcripts but enhances their translation [40].

The *in vivo* roles of the brain-specific CELFs, including CELF4, in mRNA metabolism in the nervous system remain unclear. The neuron-specific CELF3–6 nematode orthologue, UNC-75, is required for proper neurotransmission. Neurotransmission defects in *unc-75* mutants can be rescued by expression of human CELF4, suggesting that both may be involved in fine-tuning neural activity through regulation of mRNA in the nervous system [31]. Consistent with neurotransmission defects seen in *unc-75* mutants, *Celf4* deficient mice with a gene-targeted null allele have aberrant excitatory neurotransmission that leads to a complex seizure disorder [24], [25]. Adult *Celf4* null homozygotes and heterozygotes have a low seizure threshold and recurrent handling-associated convulsive seizures with severity and penetrance dependent on mouse strain background. On some strain backgrounds, homozygotes also experience non-convulsive (absence-like) seizures, showing that *Celf4* is involved in different types of seizure circuits [24], [25]. Deletion of *Celf4* from adult mice is sufficient for convulsive seizure phenotypes, whereas the absence seizure phenotype requires deletion before the end of the first postnatal week [24]. *Celf4-*deficient mice have additional neurological abnormalities including hyperactivity and hyperphagia-associated obesity [25]. Recently, a human *CELF4* mutation was described with clinical neurological and behavioral features closely resembling those of *Celf4* deficient mice, indicating that mammalian CELF4 is indeed an important regulator of neurological function [26].

CELF4 is expressed predominantly in excitatory neurons, with highest expression in pyramidal neurons of the hippocampus and the cerebral cortex. *Celf4* deletion enhances excitatory but does not alter inhibitory neurotransmission, thereby leading to increased cortical excitability and seizures in *Celf4* deficient mice [24]. An essential step in understanding the molecular mechanisms of CELF4 function in disease and in the normal state is to identify the mRNAs that CELF4 binds and regulates. Here, we used individual nucleotide resolution UV-crosslinking and immunoprecipitation (iCLIP) to identify an array of mRNAs directly bound by CELF4. We find that among the multitude of mRNAs bound by CELF4, a specific set of mRNAs encodes proteins highly enriched for function in synaptic neurotransmission, both postsynaptic and presynaptic. We also find that CELF4 preferentially binds these mRNAs in the 3′ untranslated region (3′ UTR) at a (U)GU motif that is generally known for CELF RBPs, and that CELF4 associates with large RNA granules, suggesting that it regulates neurotransmission by modulating stability, translation, and/or localization of these mRNAs. Although global changes of RNA transcripts were modest, significant shifts in RNA abundance between cell body and neuropil seen in *Celf4* null mutants combined with immunostaining localizing CELF4 to neuronal projections further supports a role for CELF4 in mRNA regulation outside of the cell body, well into axons and dendrites of CELF4-expressing cells. Immunostaining also revealed significant changes in CELF4 targets in a subset of cell types (pyramidal excitatory cells) and cellular compartments (neuropil, this study; axon initial segment, W. Sun, J. Wagnon, C.L. Mahaffey, W. N. Frankel, unpublished results) that were consistent with elevated excitability in *Celf4* mutants. Many CELF4 targets that are dysregulated are associated functionally with regulation of synaptic plasticity and of neurotransmission. Together, our results suggest that CELF4 has a central role in coordinating synaptic function in excitatory neurons.

## Results

### CELF4 binds mRNAs specifically in the 3′ untranslated region

**Figure 1:**
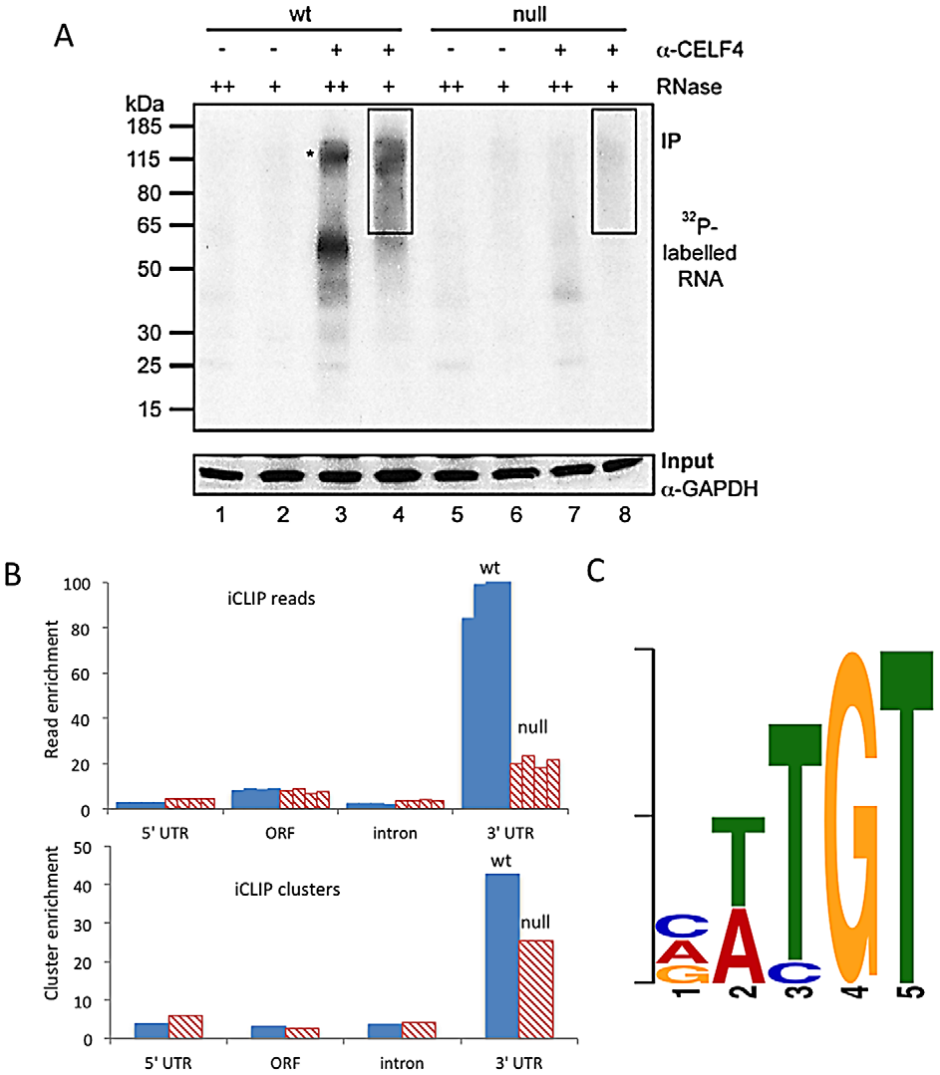
CELF4 binds mRNAs mostly in the 3′ UTR and favors an (A/U)UGU binding motif. A. Rigorous purification of CELF4-bound RNAs with iCLIP. The autoradiogram shows size-separated crosslinked protein-RNA complexes following complete digestion with high (++) or partial digestion with low (+) amounts of RNase I, immunopurification with an anti-CELF4 antibody and 5′ end radiolabeling. The boxes depict the areas on the nitrocellulose membrane from which crosslinked RNAs were purified for reverse transcription. The asterisk marks dimerized CELF4. B. Gene segment analysis showing 3′ UTR enrichment of CELF4 binding. For enrichment, the percentage of reads (upper panel) or clusters (lower panel) mapping to a particular gene segment is divided by the percentage of the genome encoding this type of segment. For read enrichment, individual replicates are shown, and for cluster enrichment, the wildtype and knockdown experiments were grouped. C. The most significant CELF4 regulatory motif discovered by comparison of significant crosslink clusters determined by CELF4 iCLIP from wildtype or *Celf4* null brain. The e-value of the top motif, as determined by DREME software, was 4.7×10^−338^.

We used iCLIP followed by high-throughput sequencing to isolate and identify RNAs bound by CELF4 in adult 129S1 mouse strain cerebral cortex and hippocampus [41] (Figure 1; File S1). Importantly, 129S1-*Celf4* null mutant brain extracts were used as a negative control. Radiolabeling of the RNA co-immunoprecipitated with an anti-CELF4 antibody shows the generally high stringency and specificity of the reaction (Figure 1A). From wildtype brain we obtained a total of 20,124,340 CELF4 iCLIP sequencing reads that could be assigned to the individual experiments (File S1). Of these, 15,625,204 could be mapped as single hits to the genome corresponding to 12,250,800 unique protein-RNA crosslinking events (iCLIP tags) that cluster into 239,218 binding sites (Table 1, File S1). In comparison, from *Celf4* null brain we obtained a total of 7,484,671 sequencing reads of which 5,328,472 had a single hit in the genome corresponding to 3,408,168 unique crosslinking events that cluster into 68,233 binding sites (File S1; Table 1). Crosslink events and clusters were strongly enriched in 3′ UTRs when CELF4 was purified from wildtype brain (Figure 1B; Table 1). Overall the binding results were reproducible, as indicated by stronger gene-by-gene correlation within each of four genotype replicates than between genotypes (File S1). To determine the occupancy of CELF4 at each binding site, we normalized crosslink counts at each site using an independent brain RNAseq dataset from the same mouse strain [ENA:ERP000614]. By using DREME software [42], comparing the clusters identified in wildtype with those identified in *Celf4* null, we identified (A/U)UGU as the favored binding motif (Figure 1C). These results provide *in vivo* support for past structural and CLIP studies of CELF1, indicating that different CELF family members recognize a very similar sequence element *in vivo* [43], [44], [45]. Interestingly, despite the very high enrichment in wildtype, in the *Celf4* null samples there was a small amount of enrichment for 3′ UTR binding (Figure 1B) and a slight preference for similar UG-rich motifs as evidenced by pentamer analysis (File S1), raising the possibility that the CELF4 antibody used for immunoprecipitation has a small amount of cross-reactivity with another CELF orthologue, as suggested previously [24].

**Table 1:** Number of unique iCLIP tags in *Celf4* wild-type and null extracts, by gene segment.

Genotype (replicate-hemisphere)	3′ UTR	5′ UTR	ORF	intron
null1 (1-R)	125,521	5,793	105,364	1,101,760
null2 (2-R)	64,749	2,638	51,158	458,846
null3 (1-L)	31,058	1,500	24,110	305,321
null4 (2-L)	58,840	2,336	42,747	461,211
wt1 (1-R)	1,016,820	7,090	201,060	1,373,130
wt2 (2-R)	1,663,030	9,603	302,963	1,621,310
wt3 (2-L)	1,299,880	7,360	232,156	1,185,560
wt4 (3-L)	735,803	4,248	128,918	679,911

### CELF4 target mRNAs comprise 15%–20% of the transcriptome and are highly enriched for synaptic functions

**Figure 2:**
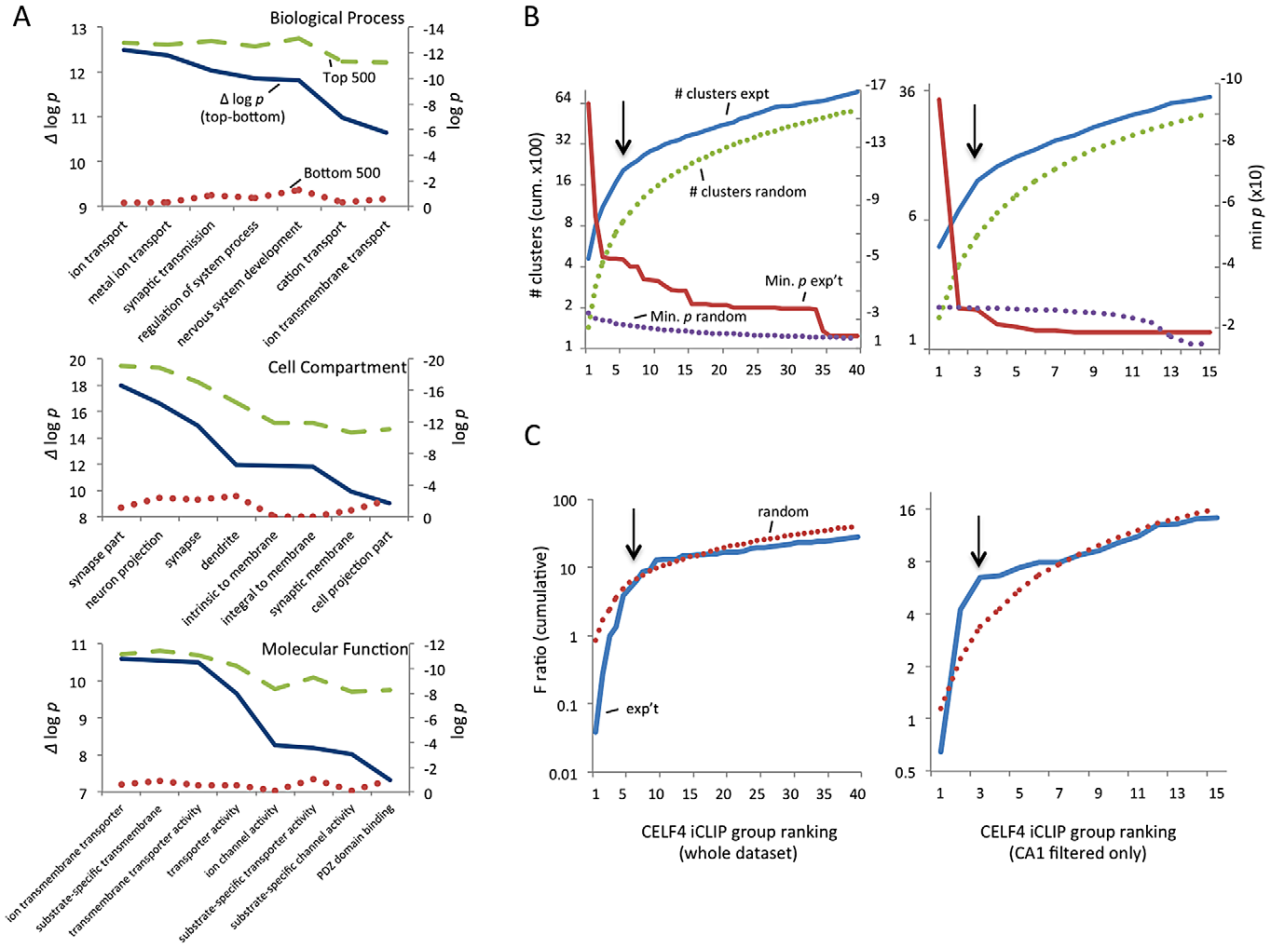
Functional annotation clustering of CELF4 mRNA targets and estimating CELF4 target binding threshold. A. Charts in this panel summarize gene ontology (GO) functional annotation clustering by “Biological Process” (top), “Cellular Compartment” (middle) and “Molecular Function” classes (bottom) for the top 500 ranked CELF4 targets (log *p* value - dashed line, green), vs. the bottom 500 ranked CELF4 targets (log *p* value - stippled line, red), and the difference between them (Δ log *p* - solid line, blue). For each class, the most significant log *p* categories are shown for either group, with no omissions. This panel illustrates how the highest-ranking CELF4 targets show strong enrichment for being associated with neurons and neuronal and synaptic functions. The VLAD tool at the Jackson Laboratory Mouse Genome Informatics website was used for this analysis (http://proto.informatics.jax.org/prototypes/vlad). The complete list of gene queries and analyses can be found in File S3. B. Enriched functional annotation clustering was exploited to approximate the threshold of significant CELF4 binding to targets based on iCLIP data. The left panel represents all 14,288 gene annotations chosen for further analysis, and the right panel a subset of 3,222 genes after filtering the larger set using a list of genes reported to represent the rodent hippocampal CA1 pyramidal neuron transcriptome [48]. For these estimates, iCLIP data were split serially by rank into 40 groups (357 genes each) for all annotations, or 15 groups (204 genes each) for the CA1 subset, each group was fed into the DAVID (v6.7) set of functional annotation tools (http://david.abcc.ncifcrf.gov) and functional annotation charts were obtained for each using the same default settings for each group. The maximum number of clusters (# clusters, exp't - solid line, blue) and minimum *p* value (Min *p* - dashed line, red) for any functional category clustered by that group was recorded and plotted cumulatively on the Y axis against each group on the X axis. As a control, the iCLIP ranking was permuted 100 times and each group fed into DAVID 6.7 and the average cluster number (# clusters, random - stippled line, green) and minimum *p* value (Min *p*, random - stippled line, purple) were similarly plotted. C. Similar to panel B, except using steady-state mRNA expression differences between *Celf4* null and wildtype brain as the indicator (F ratio – cumulative), as described in the text. For panels B and C, the black arrows indicate inflections where the correlation between iCLIP rank and enrichment (functional clustering, or change in steady-state gene expression, respectively) begin to decrease, noting the approximate binding threshold which is similar for both measures. The gene lists used in each can be found in File S3.

We first defined a maximum dataset (from RNAseq data to be used later) comprised of 14,288 genes that had 10 or more normalized reads in wildtype samples (File S2). To assess the potential of each gene as a CELF4-binding target, we ranked the genes by the CELF4 occupancy at the crosslink cluster with highest cDNA count in the wildtype dataset, after subtracting from it the highest cluster occupancy in the same gene in the *Celf4* null datasets. To initially assess any evidence for common functions in this rank-ordered list, we compared the gene ontology (GO) clustering of the top-ranking 500 genes (i.e. most likely CELF4 mRNA targets) with the bottom-ranked genes (least likely targets). The difference between these two groups was striking. For example, in the GO class “biological process”, the seven most significant GO categories for the top-ranked 500 targets showed high enrichment, with *p*-values of between 2×10^−10^ and 2×10^−13^. All but one GO category was neuron specific or related to ion transport. We noted that the next 500 highest ranked genes in the list also showed significant and specific clustering (data not shown). For the bottom-ranking CELF4 targets, no GO category had a *p* value lower than 2×10^−9^, and the lowest for any neurological-specific process was 1×10^−2^ (Figure 2A, File S3).

Because of the very strong GO category clustering of the top-ranking genes, we could use functional annotation clustering to infer a threshold for functionally relevant binding from the ranked list of CELF4 targets. This was accomplished using the DAVID analysis tool [46], [47] by “walking” down the target ranking in groups of approximately 350 genes using identical clustering criteria in each step. For this, we examined two quantitative measures: the total number of annotated clusters, and the minimum *p*-value for any category in each query group. Cumulative values were plotted against the CELF4 iCLIP ranking, and inflection points (i.e. where each line starts to “flatten”) were noted, indicating a putative threshold (Figure 2B-left, solid lines). As a control, the ranked list was permuted randomly 50 times, each replicate re-grouped, and average values plotted (Figure 2B-top, stippled lines). There were clear differences in the Y-intercepts and slope in the experimental sample compared with random – best illustrated by an approximate 300-cluster increase in Y-intercept, and a precipitous flattening of the minimum *p* value. Together, these two indicators suggested that the threshold for CELF4 target is nominally 2,000 genes (arrow), or almost 15% of 14,288 transcripts in the dataset.

CELF4 is expressed predominantly in excitatory neurons [24], but the entire cerebral cortex and hippocampus tissue extracts used for iCLIP are comprised of a heterogeneous mixture of cells. Recently a transcriptome study was done for rat hippocampus, whereby a series of filters was applied *in silico* to the experimental data to obtain a putative transcriptome for the synaptic region of a typical rodent hippocampal CA1 pyramidal neuron [48]. Although this list lacks CELF4 targets that are expressed extrasynaptically, and the CA3 region has higher CELF4 expression [24], [49], we applied this transcriptome as a filter to our iCLIP list and performed a similar analysis as above. The results were very similar to those obtained from the larger, unfiltered group (Figure 2B-bottom). A notable downward inflection in cumulative annotation clusters coinciding with a very sharp flattening in the *p*-value (arrows) together suggest that CELF4 binds around 650 of the putative CA1-specific transcripts, or about 20% of the CA1 transcriptome, which is slightly more than predicted by the unfiltered set.

Because of the potential importance of estimating a binding threshold and thus a set of genes for further analysis, we sought an independent approach. Previously it was noted that several transcripts had reduced abundance in the brain of *Celf4* null mutant mice [25]. Three of these are highly-ranked CELF4 targets: *Htr2c* (rank #64), *Snca* (#122) and *Nsf* (#374). Reasoning that altered gene expression of some or many of its targets may be a common feature of CELF4 deficiency, we then compared genotype-dependent changes in transcript expression against the CELF4 iCLIP ranking. A custom microarray of *Celf4* null and wildtype whole brain mRNA was done and differential expression was plotted against the CELF4 iCLIP ranked groups (Figure 2C). Similar to the results from functional annotation clustering, the experimental curves for differential gene expression had a steeper rise followed by more rapid diminution when compared to randomly permuted data - this was evident for all 12,016 positively expressed genes in this array, and was particularly notable in the putative CA1 transcriptome subset (Figure 2C-right). The apparent threshold was remarkably similar to that obtained from functional annotation clustering.

In summary, although there is no *a priori* way to directly define the threshold for significant *in vivo* binding in iCLIP experiments, two independent measures—functional annotation clustering, and altered transcript abundance—suggest that CELF4 directly regulates at least 2,000 mRNA targets, corresponding to a large fraction of the excitatory neuron transcriptome.

### CELF4 co-sediments with very large RNA granules and is present in neuronal soma and dendrites

**Figure 3:**
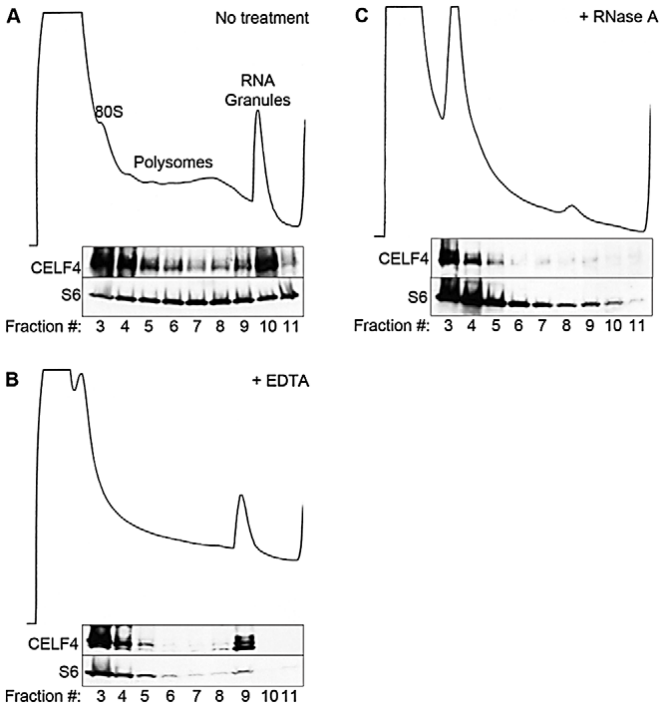
CELF4 cosediments with polysomes and large RNA granules. A. Cortical brain tissue lysates from 4-week old mice were fractionated on 15–55% linear sucrose gradients. Fractions were collected and analyzed by immunoblot with antibodies against CELF4 and ribosomal S6 (S6) protein. Lysates were treated with either EDTA (B) or RNase A (C) in parallel. Sedimentation is show from left to right, with the positions of monosomes (80S), polysomes, and RNA granules indicated.

The enrichment of CELF4 binding in the 3′ UTR suggests a role in control of translation, stability, and/or localization. Cytoplasmic ribonucleotide-protein complexes (RNPs), including translating polyribosomes and very large neuronal RNA granules (processing bodies, stress granules, and transport granules harboring translationally silenced particles) control the fate of mRNAs in response to a cell's needs [14], [50], [51], [52]. These very large granules can be distinguished from polyribosomes by using sucrose density gradient fractionation, referred to henceforth as polysome fractionation [52], [53], [54]. To assess the fate of CELF4-bound mRNAs upon CELF4 loss, we performed polysome fractionation on cortical brain lysates from 4-week old wildtype and *Celf4* null mice to separate translating polyribosomes from large RNA granules and examined CELF4 distribution in individual fractions by immunoblotting. Even though CELF4 was found in all fractions, it was particularly prominent in the very high density RNA granule fraction pool (Figure 3A). This presence was independent of ribosomes, as indicated by the inability of EDTA to release CELF4 from the granular fractions (Figure 3B). This result stands in contrast to other RBPs, such as FMRP, that interact directly with ribosomes and shift upon EDTA treatment [55], [56]. Treatment of polysomes with RNase A, however, destabilized RNA granules, causing a corresponding loss of CELF4 from the very high density fractions (Figure 3C).

**Figure 4:**
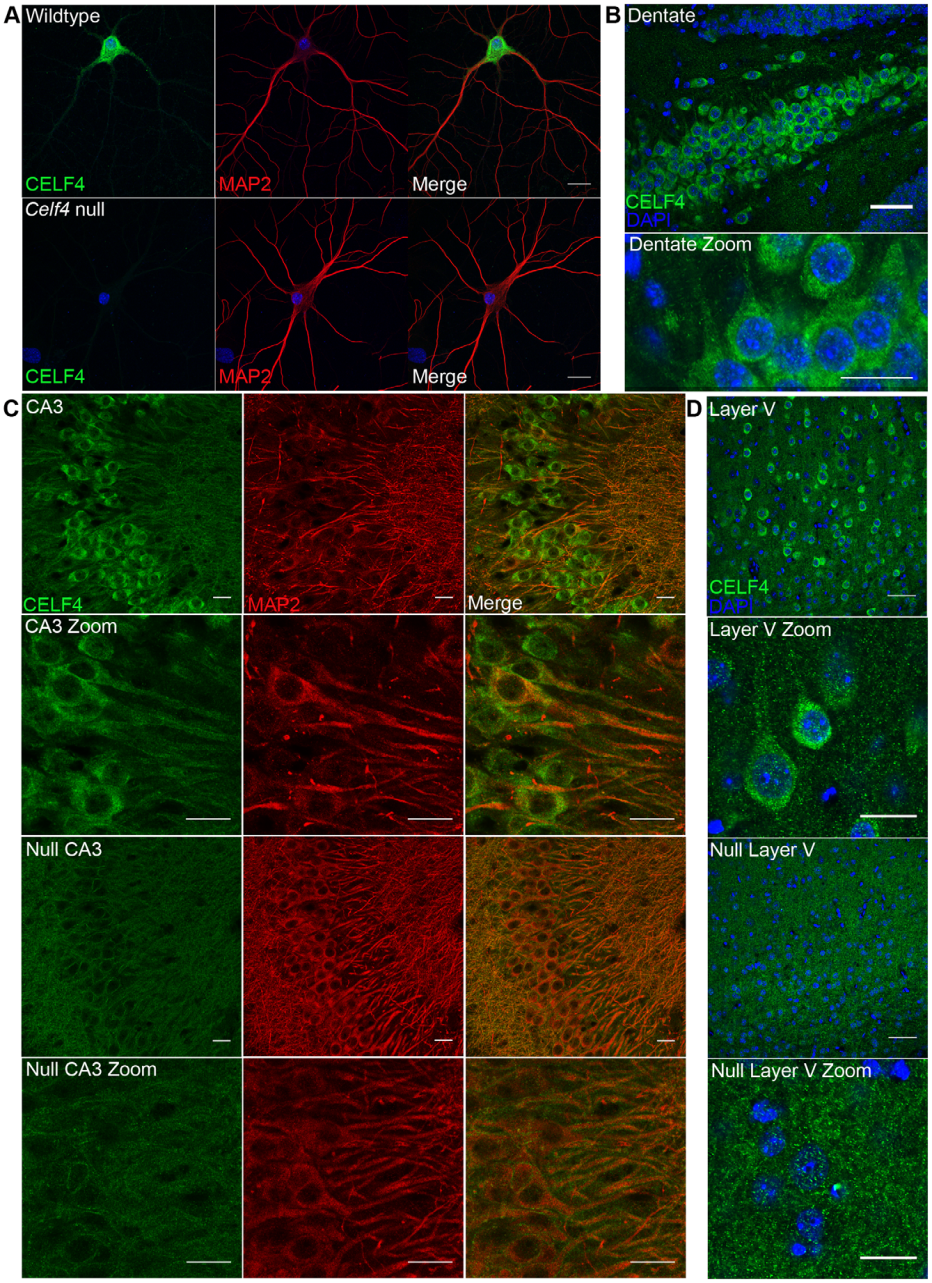
CELF4 protein is localized in soma and in neuronal projections. A. Wildtype DIV14 cultured primary hippocampal neurons were examined for CELF4 protein localization by immunostaining with antibody against CELF4. Neuronal projections were stained with antibody against MAP2. Scale bar 20 µm. B–D. Sections from adult wildtype and *Celf4* null mouse brains were examined for CELF4 protein localization by immunostaining with antibody against CELF4. In coronal cryosections, high CELF4 expression is seen in hippocampus, including the dentate gyrus (B), and in cortical layer V pyramidal neurons (D), which show CELF4 localization in soma and apical dendrites (D - top two panels). In sagittal vibratome sections, CELF4 localizes to soma and into dendrites in the CA3 region of the hippocampus as shown by colocalization with MAP2 (C – top two panels). The CELF4 antibody is specific as no signal above background is seen in *Celf4* null hippocampal or cortical layer V cells (C,D - bottom two panels). Nuclei were stained with DAPI. Scale bar 20 µm.

In addition to the cytoplasm of the soma, RNA granules might exert their activity in neuronal projections, where large RNA granules have been shown to fulfill roles in mRNA transport and localized translation [14]. CELF4 expression was previously found strongest in the soma of excitatory neurons [24], but those studies lacked resolution to determine whether it was also in dendrites or axons of individual neurons. By immunostaining we detected CELF4 in the soma, with a predominant cytoplasmic pattern, as well as in proximal processes of dissociated hippocampal neurons (Figure 4A). In the projections, punctate immunostaining for CELF4 was visible (Figure 4A). Similarly, in mouse brain, where the antibody reacted specifically with CELF4 as evidenced by immunostaining of cerebral cortex and hippocampal CA3 in wildtype but not *Celf4* null mutants (Figure 4B–4D), CELF4 was present in the cell bodies and proximal processes of the dentate gyrus (Figure 4B), hippocampal region CA3 (Figure 4C), and cortical layer V pyramidal cells (Figure 4D). In the hippocampus, CELF4-immunopositive projections extended visibly into the neuropil (Figure 4C). However, CELF4 is probably not present at the nerve terminal itself, as evidenced by its absence from synaptosome fractions (Figure S1). Together with previously published data, these new findings suggest that CELF4 exerts its role in posttranscriptional regulation of RNAs at multiple sites throughout the excitatory neuron

### CELF4 deficiency affects the abundance and distribution of its target RNAs

Next, we studied the fate of CELF4 target transcripts in CELF4 deficient mice. First, a custom microarray was used to simultaneously examine alterations in steady-state mRNA abundance and alternative splicing in *Celf4* null compared to wildtype adult brain. Investigating the alternative splicing defects is of interest since both CELF1 and CELF2 are involved in splicing and at least one prior study suggested that CELF4 might be as well [28]. However, consistent with the strong enrichment of CELF4 binding in 3′ UTR regions, we found very little evidence for a significant role of CELF4 in splicing, since we were able to validate small splicing changes in only five genes: *Ank2*, *Cacna2d2*, *Cadps*, *Grm5* and *Ppp3ca* (data not shown). In contrast, at the full transcript level, 628 (5.2%) of 12,016 genes did show a highly significant difference in expression, using an experiment-wise significance threshold of p<0.05 (File S4). 144 (23%) of these were among the top 2,000 CELF4 targets identified by iCLIP (File S4). The expression changes, however, were of a modest nature with the maximum difference corresponding to a 40% change, consistent with results reported previously for genes now known to be CELF4 targets [25]. Although more than half of these CELF4 targets showed evidence for increased expression in *Celf4* null brain (84 *vs.* 60 with decreased expression, respectively), the average fold change was significantly greater for targets with decreased expression (*p*<0.001, t-test). It is likely, however, that many more than these 144 CELF4 targets actually show “real” expression differences, when compared with the rest of the transcriptome. For example, there was still a significant difference in the average differential expression between the remaining top priority CELF4 targets and the remaining 9739 genes on the microarray (*p* = 0.0008, t-test), with again, a tendency for them to be expressed at lower levels in *Celf4* null mutant (p<0.001, t-test).

### Validation of CELF4 target gene expression changes in *Celf4* null mice

**Figure 5:**
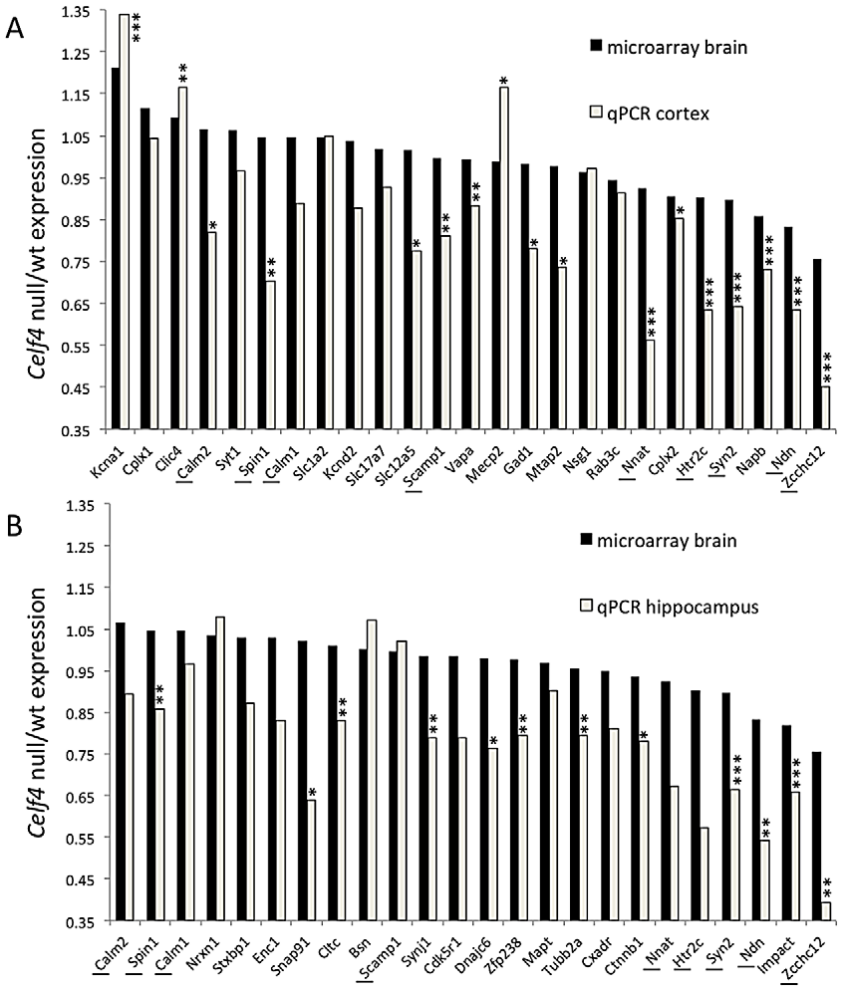
Validation of CELF4 genotype-dependent abundance changes of select CELF4 target mRNAs. The white column in each panel shows relative fold change (expressed as 2^ΔΔCt^ –Methods) between *Celf4* null and wt for dissected cerebral cortex (panel A), or hippocampus (panel B), respectively by quantitative real-time RT-PCR (qPCR). In both panels filled columns show the ratio of normalized average fluorescence between *Celf4* null and wt from the whole brain microarray. For qPCR data, statistical significance was determined using Student's |t| test: *p<0.1, **p<.05, ***p<0.01. Underlined gene symbols highlight genes tested in both tissues. The ΔΔCt data for qPCR and subset of microarray data can be found in File S4.

**Figure 6:**
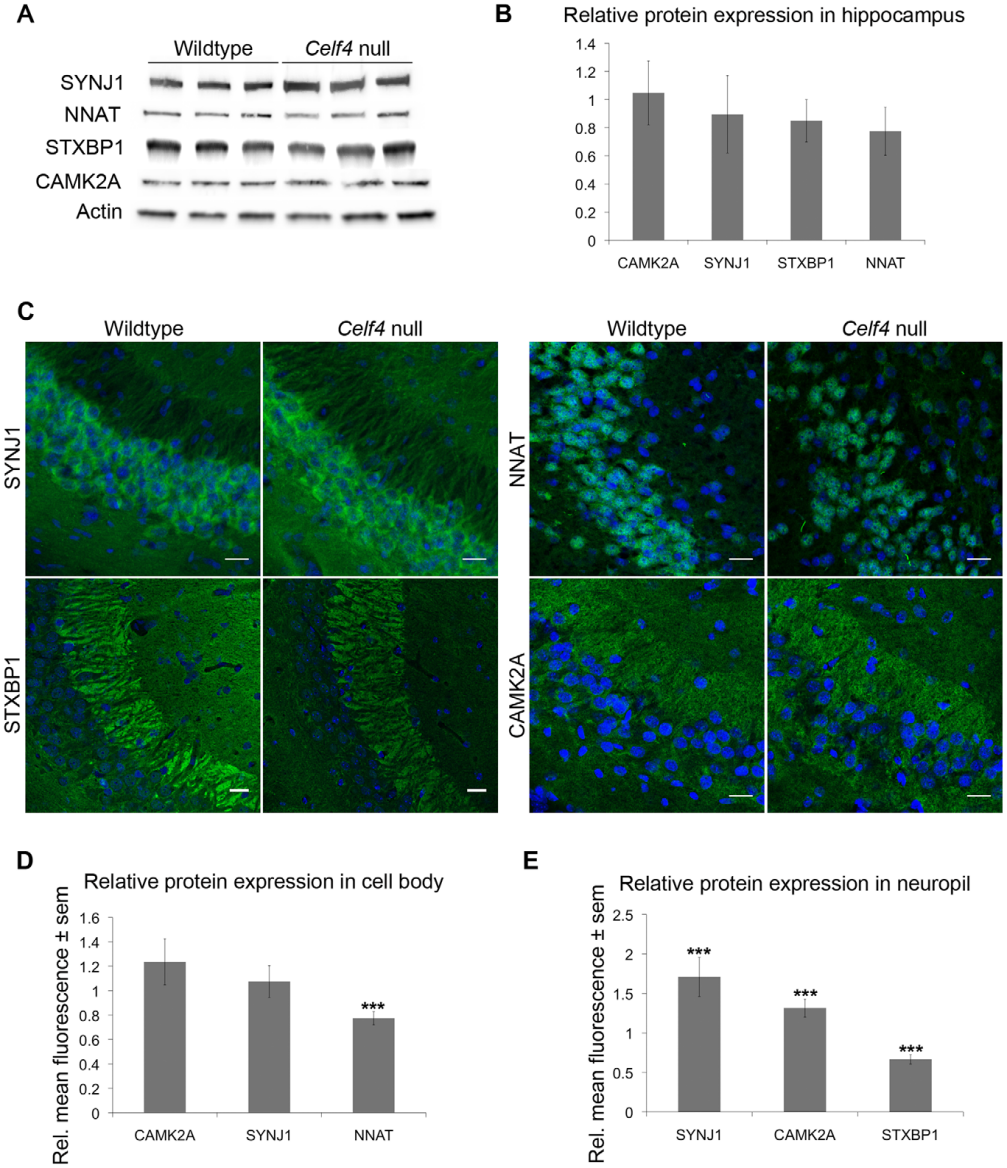
*Celf4* genotype-dependent whole cell versus subcellular expression for four CELF4 targets. A, B. Protein abundance in wildtype and *Celf4* null hippocampal tissue extracts were examined by quantitative western blotting. Representative immunoblots are shown; each protein was assessed in extracts from three mice of the same genotype. Immunoblots were visualized with chemiluminescence and signal was captured with a cooled CCD camera. B. Quantification of protein abundance was performed using ImageJ. Each sample was normalized to actin. Relative mean OD values of the *Celf4* null samples compared to wildtype samples are shown with standard deviation. C–E. Sagittal sections from wildtype and *Celf4* null mutant mouse brains were examined for CELF4 target protein expression using immunostaining. C. Representative images from the CA3 region of the hippocampus is shown. D. For cell body, fluorescence was quantitated in ImageJ by measuring the mean intensity for each positive cell body. Background was subtracted and average mean fluorescence for *Celf4* null and wildtype were calculated. Data are presented as mean fluorescence of *Celf4* null relative to wildtype ± sem. E. For dendrites, fluorescence was quantitated in ImageJ by measuring the mean intensity for each positive dendrite in the stratum radiatum or positive region in the stratum lacunosum-moleculare. Background was subtracted and average mean fluorescence for *Celf4* null and wildtype were calculated. Data are presented as mean fluorescence of *Celf4* null relative to wildtype ± sem. For D and E, statistical significance was determined using Student's |t| test: ***p<0.01.

We next sampled steady-state expression of 25 CELF4 targets each in cerebral cortex and hippocampus by quantitative real-time RT-PCR (qPCR) in biological triplicate, including representative targets that either were increased or decreased in the *Celf4* null brain microarray, were high ranking iCLIP targets, whose expression was examined previously in *Celf4* mutants [25] or that are associated with synaptic regulatory functions based on findings described below. The qPCR results generally correlated with the microarray (Figure 5A-cortex; Figure 5B-hippocampus), and with one exception (*Scamp1*), for 9 targets that were examined in both tissues the relative trend was the same. Previously, steady-state, whole cell protein expression was examined by immunoblot and it correlated well with RNA for four genes that we know now are targets (*Htr2c*, *Nsf*, *Syn2*, *Snca* [25]). Because we know now from the comprehensive iCLIP screen that many CELF4 targets encode proteins that function in neuronal projections, for a few targets we compared whole cell protein expression (by western blot) with subcellular expression in hippocampus where it is relatively easy to distinguish soma from neuronal projections by immunostaining. Whole cell expression changes were modest, but trended towards downregulation consistent with the mRNA levels (Figure 6A and 6B). Subcellularly, some targets such as NNAT and STXBP1 followed this trend and were lower in cell body and neuropil, respectively (Figure 6C–6E). Interestingly, however, for two CELF4 targets, SYNJ1 and CAMK2A, that could be evaluated in both cell body and neuropil, protein levels were significantly increased specifically in neuropil (Figure 6E). Altogether while these and previous mRNA and protein studies confirm that CELF4 loss does result in altered abundance of many targets with a general trend towards downregulation at the whole cell level, some targets appear to exhibit pronounced differences in relative abundance between cell body and neuronal projections.

### *Celf4* genotype-dependent shifts of CELF4 targets in polysome fractions and in the cell body-neuropil axis

**Figure 7:**
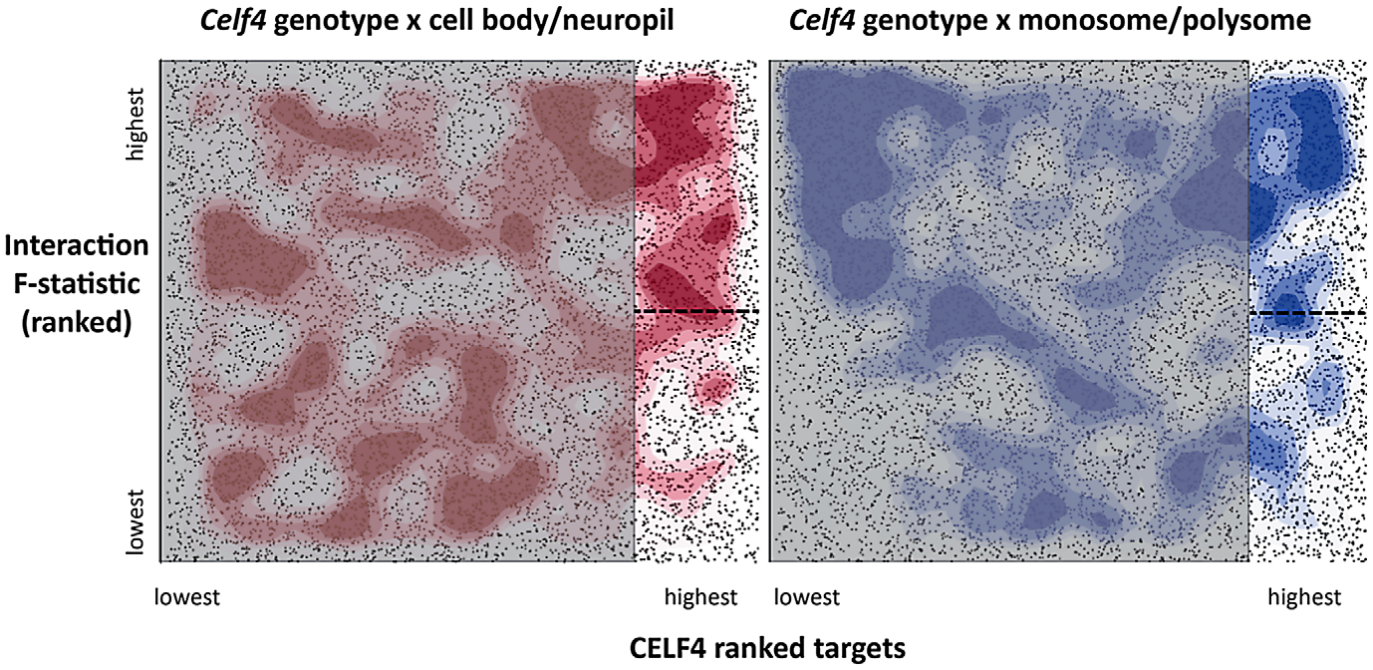
Visualization of *Celf4* genotype-dependent shift of CELF4 target mRNAs between polysome fractions and along the cell body/neuropil axis of CA1 hippocampal neurons. Each chart shows the entire space examined in interaction ANOVA models described in the text and summarized on Table 2, for the hippocampal CA1 cell body vs. neuropil experiment (left panel) and the monosomes vs. polysomes experiment (right panel). Each square shows individual genes (dots), plotting the rank of their iCLIP occupancy score (X axis) against the rank of their interaction F-statistic (Y axis). Less likely CELF4 targets (below the top 2,000 occupancy scores) are de-emphasized by masking, and the most likely 2,000 CELF4 targets are unmasked on the right of each square. Density contouring (applied using a standard graphing in JMP software) reveals where CELF4 target rank is most associated with differential expression. The dashed line at the right of each divides the most likely CELF4 targets in half by F-statistic score—these two halves are compared to each other for the analysis shown in Figure 8. We note that the monosomes vs. polysomes experiment did show strong contouring in the corresponding upper-left quadrant. These entries correspond largely to mitochondrial mRNAs or nuclear or mitochondrial mRNAs that encode ribosomal proteins (data not shown).

To more systematically study the fate of CELF4 targets at the subcellular level, we examined their distribution a) between hippocampal cell body and neuropil and b) among polysome fractions by using RNAseq, comparing *Celf4* null mice to wildtype for each experimental treatment. First, we prepared polysomes from cerebral cortex and hippocampus and pooled individual fractions into three groups: monosomes (low density), polysomes (moderate-heavy density), and very large RNP and RNA granules (very high density). This was done to allow for two comparisons of transcript content potentially relevant to CELF4 function: monosomes vs. polysomes, and RNA granules vs. monosomes/polysomes. Second, to examine whether there was evidence for a genotype-dependent shift in the subcellular distribution of CELF4 targets we chose the CA1 region of the hippocampus because of the relative ease with which its cell bodies and neuropil can be dissected. The polysome-fractionated and the CA1-derived mRNA were subjected to high-throughput sequencing followed by reference genome alignment and data normalization. For the analysis of the dependence of mRNA levels on *Celf4* genotype, we first obtained F-statistics for genotype-by-treatment interaction in linear models by analyzing an ANOVA of ranked, normal quantile-transformed data (File S5 for the whole dataset; File S6 for the putative CA1 transcriptome subset). In a follow-up analysis, we examined the relationship between expression and CELF4 iCLIP ranking (Table 2). Although genotype-dependent transcript abundance between RNA granule vs. monosome plus polysome together had no apparent correlation with CELF4 target status (F, 2.18; ns), a highly significant correlation was seen for monosomes vs. polysomes (F, 24.29; p<0.0001), and for CA1 cell body vs. neuropil (F, 12.46; p<0.001). Similar results were obtained regardless of whether we used the entire dataset, or after filtering for the putative CA1 transcriptome (Table 2). The relationships between CELF4 target ranking and *Celf4*-dependent transcript abundance for each model are visualized in Figure 7, where the entire interaction space is depicted by a square containing individual genes (dots), density contouring emphasizes where CELF4 target ranking is associated with differential abundance, and the highest ranking (top 2,000) CELF4 targets are unmasked, with high and low abundance changes separated by a dashed line. These results show that in the dissection experiment especially, much of the signal comes from the highest-ranked CELF4 targets.

**Table 2:** *Celf4* genotype-dependent shifts (“Interaction”) of CELF4 target mRNAs.

	All genes		CA1 filtered		
Experiment	Fstat	Adj. pval	Fstat	Adj. pval	Model
Monosomes vs. polysomes	65.8		16.0		Experiment
	6.2		16.7		*Celf4* genotype
	53.0	*p*<0.0001	21.6	*p*<0.0001	Interaction
RNA granules vs. mono&polysomes	4.2		15.9		Experiment
	103.1		28.5		*Celf4* genotype
	2.3	NS	1.0	NS	Interaction
CA1 neuropil vs. cell body	0.6		2.5		Experiment
	3.8		0.1		*Celf4* genotype
	12.5	*p*<0.001	10.3	*p*<0.001	Interaction

### Functional annotation clustering reveals a selective shift of CELF4 targets in the neuropil of CELF4-deficient neurons

**Figure 8:**
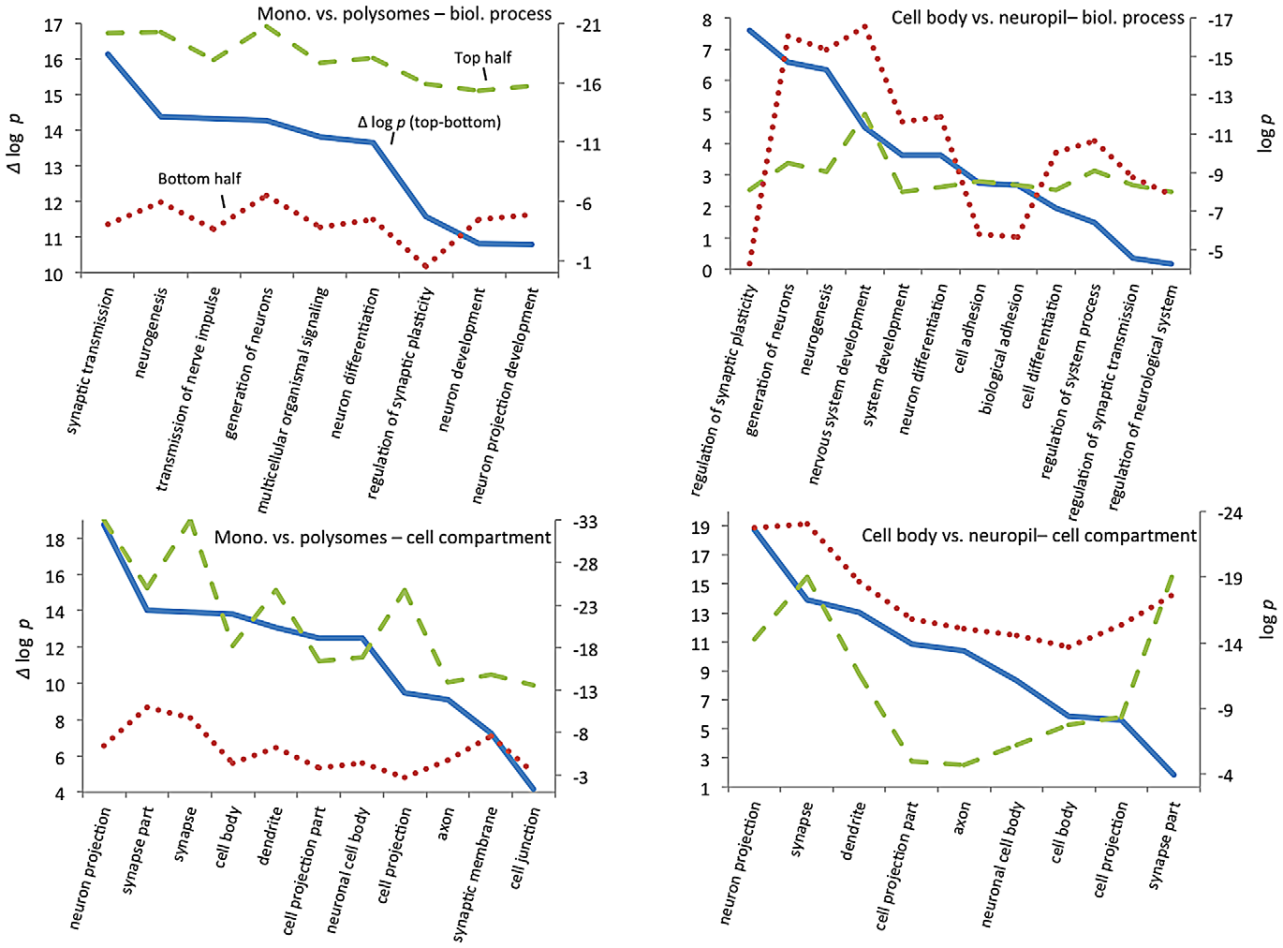
GO functional annotation clustering of CELF4 targets. These charts summarize gene ontology (GO) functional annotation clustering by “Biological Process” (top panels) and “Cellular Compartment” (bottom panels) for the most differentially expressed CELF4 targets compared with the least differentially expressed - as derived from the interaction ANOVA models (Table 2; Figure 7) for the monosomes vs. polysomes experiment (left panels) or the hippocampal CA1 cell body vs. neuropil experiment (right panels). The format and approach to construction of these charts were the same as for those shown in Figure 2A. The analysis illustrates how differentially expressed CELF4 targets in the monosomes vs. polysomes experiment cluster very significantly across many neuronal biological processes and cell compartments, whereas only a subset of CELF4 targets are associated with biological processes and sites that are differentially expressed between cell body and neuropil. The gene lists and VLAD output for all data, as well as the list of 142 enriched genes as derived from this analysis, may be found in File S8.

To explore how CELF4 targets drive these highly significant genotype-dependent interactions, we examined functional clustering of “GO biological process” categories, by comparing CELF4 targets that had the most significant differential abundance scores (as Δ log *p*) to those with the lowest, as divided along the interaction F-statistic median (i.e. dashed line in Figure 7). For monosomes vs. polysomes, there was an overwhelmingly higher degree of clustering among CELF4 targets with the most differential abundance (Figure 8-left; all results may be found in File S7). “GO biological process” terms were dominated by only highly significant neurological clusters including “synaptic transmission”, “neurogenesis” and other broad aspects of neuronal or synaptic function (Figure 8-top left). Similarly, monosomes vs. polysomes by “GO cell compartment” terms also revealed very significant differential scores, led by “neuron projection” and “synapse part” but covering all parts of the neuron (Figure 8-bottom left). In stark contrast, however, the CA1 cell body vs. neuropil experiment gave a different set of results. Most of the functional clustering was associated with CELF4 targets that had the least amount of differential abundance between cell body and neuropil (Figure 8-right). Thus, while generally the same GO categories showed the most significant clustering, all but two “GO biological process” terms (“regulation of synaptic plasticity” and “cell adhesion”) and only one of the top “GO cell compartment” terms (“synapse part”) exhibited more clustering among differentially abundant CELF4 targets.

Together these results reveal a model whereby CELF4 regulates a large number of genes involved in neuronal function across the cell, by limiting the degree to which transcripts are available for translation. Furthermore, a subset of CELF4 targets that are differentially abundant between cell body and neuropil are implicated in synapse-specific processes, led by the GO category “regulation of synaptic plasticity” and including “regulation of synaptic transmission.”

**Figure 9:**
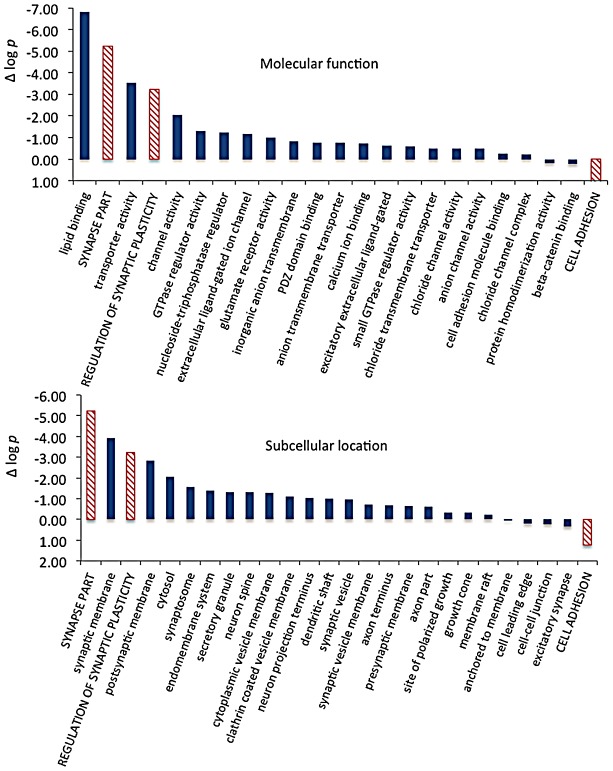
Significance and direction of effect for synaptic CELF targets enriched between cell body and neuropil. This figure, together with Table 3, considers the subset of 142 CELF4 targets selectively enriched for differential expression between hippocampal CA1 cell body vs. neuropil, as derived from the GO categories “Regulation of synaptic plasticity”, “Synapse part” and “Cell adhesion”, from Figure 8. The relative significance is shown for the three proxy categories (non-solid columns) and various subcategories (solid columns) of molecular function (panel A) and subcellular location (panel B), using the difference in the log *p* value derived from Fisher's Exact test for each (Δ log *p*), as an indicator (Y-axis). Categories were selected based on having at least 12 CELF4 targets in each, and also only one category is shown if closely-related GO categories had the same members in them (e.g. “transporter activity” was shown but not “ion transporter activity”, “transmembrane transporter activity” which had the same members). For molecular function, the largest effect is seen for proteins that associate with “lipid binding”, transporter activity” and “channel activity”, with the next category being almost an order of magnitude less significant. For subcellular localization, the subcategories that had the largest effect were “synaptic membrane”, and “presynaptic membrane”, although various other structures were almost as significant. The full list of genes, categories, sample sizes and expression data can be found in File S8.

We then extracted the 142 gene annotations from the three proxy GO categories most enriched for differentially abundant CELF4 targets (“regulation of synaptic plasticity” and “synapse part” —82 genes total; “cell adhesion” —60 genes), in order to examine the direction of their effects and to begin to draw inferences about mechanisms that CELF4 regulates at the synapse (Figure 9, Table 3). For this, we reclassified the 142 genes as being associated with a variety of subcellular localization and molecular function GO categories. For each category we examined the relative abundance and the direction of effect between *Celf4* null and wildtype genotypes in the two experiments (monosomes vs. polysomes, and cell body vs. neuropil) by using contingency table analysis (Fisher Exact test *p*). We also compared effects between experiments (by comparing Δ log *p*). For categories that changed the most in *Celf4* null mice, the tendency was toward higher abundance in polysomes compared with monosomes, and in neuropil compared to cell body (all data from all categories can be found in File S8). Figure 9 summarizes the results, showing the three proxy categories (stippled bars), and the most significant subcategories (solid bars) by molecular function (top panel) and subcellular localization (bottom panel). All but one category showed a more negative log *p* ratio, indicating that in *Celf4* null mice these transcripts were more abundant in neuropil than in the cell body. For the most significantly altered CELF4 targets, directions of effect were visualized in Table 3.

**Table 3:** Number of genes in select enriched GO categories: Relative abundance in *Celf4* null versus wild type.

		Polysomes		Neuronal axis	
Category	Direction	*Celf4* null poly/mono	wt poly/mono	*Celf4* null np/cb	wt np/cb
Reg. synapt. plasticity	>1	16	13	19	7
	<1	5	8	2	14
Cell adhesion	>1	41	23	44	31
	<1	25	43	22	35
Synapse part	>1	38	29	56	30
	<1	30	39	12	38
Transporter activity	>1	7	6	22	9
	<1	18	19	3	16
Channel activity	>1	4	4	14	6
	<1	12	12	2	10
Synaptic membrane	>1	20	18	33	18
	<1	18	20	5	20
Postsynapt. membr.	>1	14	14	26	15
	<1	16	16	4	15

Molecular function categories reveal that most CELF4-regulated activity at the synapse is in membrane-associated proteins, including transporters and ion channels (Figure 9A). Assessment of subcellular localization categories (Figure 9B) suggests that most CELF4-regulated activity is at the synapse itself, although a fairly even trailing of other categories belies the possibility that CELF4 regulates a wide variety of molecules in many aspects of synaptic transmission. Indeed, while other, more specific categories may be revealing (e.g. “glutamate receptor activity”) the sample sizes for these were often too small to draw strong inference. However, the power of this type of analysis will only be enhanced as more experimental evidence accumulates for the functions of these genes.

In summary, while CELF4 deficiency results in widespread but modest differential steady state expression of its targets, with a significant shift towards polysomes, a subset implicated in regulation of synaptic plasticity and transmission increases in abundance and shifts towards polysomes in the neuropil.

## Discussion

In either the null or haploinsufficient genetic state, CELF4 deficiency in mice causes a complex neurological syndrome with epileptic seizures as a prominent feature but which also includes hyperactivity and obesity in aging males [24], [25]. It is known that *CELF4* is at the center of human del(18q) syndrome interval (discussed in [26]), where it is one of several genes that could be the cause of neurological symptoms of these patients, including seizures. Consistent with the murine phenotypes, a patient was described very recently with many of these features (seizures, borderline intelligence, behavioral disabilities and obesity) carrying a translocation that specifically disrupts the *CELF4* gene, thus directly implicating CELF4 in several key features of del(18q) disease. It is not yet known whether more subtle *CELF4* variants are associated with more common synaptic diseases, such as idiopathic epilepsy, ASD and various other non-degenerative neurological conditions, but with the increasingly popular applications of high throughput DNA sequencing it seems likely that this question will be answered before too long.

To understand the functions of CELF4 *in vivo*, we used iCLIP to identify CELF4-bound mRNAs. The main finding of this aspect of the study is that CELF4 regulates translation and local abundance of a vast set of mRNAs, and its primary role is not in regulation of alternative splicing as was proposed from earlier *in vitro* studies [28]. Several lines of evidence strongly support this claim: (i) CELF4 binds specifically within the 3′ UTR known to harbor elements for translational control and localization, and via a binding motif that is similar to that of other CELFs, (ii) using a splicing microarray only very minor splicing defects in a small number of genes were detected, (iii) CELF4 co-sediments with very high density RNP particles, (iv) CELF4 is located in the cytoplasm of the soma as well as in neuronal projections, and (v) compared to the splicing annotation differences between genotypes which were minimal, considerably more alterations were detected in *Celf4* null mutants when assessing the distribution of mRNAs between mono- and polysomes, and between cell bodies and neuropil.

We took a novel approach towards estimating the number of true CELF4 binding targets from iCLIP. First, we established a baseline of background binding by using extracts from both wildtype and *Celf4* null mice, and observed the very strong specificity for the 3′ UTR. After deriving a ranked list of targets by considering site occupancy and background, we observed both striking functional annotation clustering as well as a broad effect of *Celf4* genotype on steady-state expression among the highest ranking targets when compared with the lowest ranking targets. We then exploited these two external measures of function—gene ontology annotation clustering and expression change—as indices to infer biological “meaning” from the ranking, by walking-down the list from highest to lowest ranked targets, and determining the inflections where the rate of clustering (or gene expression change) began to diminish. Data permutation assured that these inflections were not random. From a set of over 12,000 transcripts corresponding to many cell types (e.g. from whole brain extracts), including many excitatory neurons most of which express CELF4, or over 3,000 genes for a less heterogeneous source (by applying a recently proposed CA1 neuron transcriptome as a filter [48]), this threshold corresponds to somewhere between 15% and 20% of the transcriptome. For most of our analysis, we took the top 2,000 ranked targets as a nominal threshold for defining the CELF4 regulome, although this may be conservative as many lower ranking genes are also likely to be targets. We do not know enough yet about the relationship between the number of binding sites or affinity, and biological outcome to be any surer.

In our study, the identification of these numerous mRNA targets combined with the detection of changes in expression, translation and localization of mRNAs in *Celf4* null mutants all serve to underscore the notion that alterations of many different molecular pathways contribute to complex neurological diseases. Merely considering seizures as a phenotype, from our current and previous studies we can now infer that normal CELF4 regulation of its mRNA targets is important for early postnatal development, else absence seizures may arise, and also during adulthood, whence somatic CELF4 deletion is sufficient to cause seizures even after normal development [24]. Thus, although for this study we focused on the role of CELF4 in regulating molecules associated with function at the synapse, by extension the myriad of other properties of CELF4 targets (as suggested by functional annotation clustering) implies that CELF4 very likely is involved in coordinating a wide spectrum of neuronal functions.

With a set of targets that cover many different aspects of synaptic function, together with seizures as the major phenotype of *Celf4* deficient mice, we suspected that a general function of CELF4 might be as a regulator of the synaptic response in excitatory neurons, i.e. where it is predominantly expressed. Regulation of homeostatic plasticity and synaptic scaling is emerging as a theme for neurological disorders, including synaptic excitation [57], and it is a plausible major component for genetically complex neurological diseases like epilepsy or autism, as disruptions in regulating homeostasis would be expected to lower the threshold for other insults—genetic or environmental—leading to wider dysregulation at the circuit level. It will be very interesting in future experiments to examine whether CELF4 is involved in homeostasis or in other forms of synaptic plasticity, such as LTP.

In a single genetic model, *Celf4* mutants provide a means to evaluate both an instigating insult and a general inability to respond to it. Thus, in a companion study (W. Sun, J. Wagnon, C.L. Mahaffey, W. N. Frankel, unpublished results), using primarily an electrophysiological approach supported by genetics, we determined that a relatively modest increase in the amount of the sodium channel Na_v_1.6 (encoded by *Scn8a*) observed in the axon initial segment where Na_v_1.6 initiates action potentials in excitatory neurons [58], [59], [60], leads to a marked upregulation of persistent sodium current and intrinsic excitability of cortical layer V pyramidal neurons. Merely reducing the dosage of wildtype *Scn8a* by half completely blocks the effect of *Celf4* deficiency on seizure threshold, betraying the very dominant role that Na_v_1.6 has in the excitatory response. Importantly, since CELF4 is expressed only in a few inhibitory neurons [24], its effect on Na_v_1.6 would be mainly in excitatory neurons without increasing the excitability of inhibitory circuits. Even in this condition, it is possible that a modest increase in Na_v_1.6 might normally be tolerated, but combined with an impaired regulatory response as implied by our genomic studies, *Celf4* mutant excitatory activity would be out of control – hence recurrent seizures.

In accord with our finding that CELF4 is involved in localization and translational regulation of mRNAs, we found it to co-sediment with large RNA granules. Thus, our observations are consistent with a molecular mechanism for CELF4 that is analogous to that described for the CELF orthologue Bruno in the regulation of its target transcript *oskar*, which requires spatially restricted expression in the *Drosophila* ovary epithelium. Bruno mediates translational repression of *oskar* mRNA by binding in the 3′ UTR and either interacting with eukaryotic initiation factor 4E (eIF4E) and the 4E-binding protein Cup (eIF4ENIF1) to inhibit cap-dependent translation [61], [62] or by forming a very large RNP, or “silencing particle”, containing *oskar* mRNA oligomers that is inaccessible to translation machinery [61]. Spatially restricted gene expression is also a critical process in neurons as lasting activity-dependent synaptic plasticity requires rapid, local protein synthesis in neuronal projections in response to synaptic stimuli [14]. In neuronal projections, mRNAs populate different types of RNA granules, large RNPs that include transport granules, translating polysomes, stress granules, and processing bodies [14], [50], [51], [63]. Our results indicate that CELF4 is part of a novel large RNP particle of as yet unknown composition. Going forward, it will be important to dissect its components and investigate whether CELF4 participates in mRNA oligomerization into silencing particles similar to those formed by Bruno. It is not, however, a straightforward leap from *Drosophila* ovary to the mammalian nervous system. For example, expression of eIF4ENIF1, the orthologue of the Bruno-interacting protein Cup, is low in the mammalian brain. It is also likely that CELF4 and its bound mRNAs will dynamically interact with other types of RNPs in response to synaptic signaling. Given that CELF4 binds a subset of mRNAs highly enriched for synaptic function, insights into the CELF4 RNP should expand our current understanding of neuronal RNA granules and local regulation of RNA translation in excitatory neurons.

The RNA-binding proteins (RBPs) that form RNA granules are emerging as causal agents of disease in complex neurological and neuropsychiatric disorders, such as epilepsy, ASD, schizophrenia and ID. The high incidence of comorbidity between these conditions suggests that common pathological mechanisms underlie these disorders, and many recent studies indicate synaptic dysfunction, including abnormal synaptic protein levels, defects in synapse formation, and impaired synaptic homeostasis, as a unifying component [14], [64], [65], [66]. For example, FMRP regulates activity-dependent translation in dendrites by stabilizing mRNAs at synapses for local protein synthesis [67], [68]. FMRP loss leads to dysregulation of local RNA translation, aberrations in dendritic spine morphology, and altered neuronal excitability that increases seizure susceptibility [69], [70]. Recently, the subset of RNAs bound by FMRP was identified, and it included many molecules related to synaptic function and linked to autism [55], [71]. When cross-referenced to the curated list of potential ASD candidate genes in AutDB [72], 17% of the candidate autism genes are represented in the FMRP-bound set. Although CELF4 shares approximately 30% of its RNA targets with FMRP, a much higher percentage of autism candidate genes, over 30%, are represented in the CELF4-bound set. For the 142 CELF4 targets that show the most differential expression between cell body and neuropil, approximately 13% are found in AutDB (representing ∼5.6% of candidate genes). Therefore, it is reasonable to suspect that variants in human *CELF4* could contribute not only to epilepsy, but also—and maybe more so—to ASD and ID, which is corroborated by the spectrum of symptoms seen in del(18q) syndrome and specifically in the recent patient with the *CELF4* translocation [26].

## Methods

### Animal care, procedures, and genotyping

All animals were fed standard National Institutes of Health diet containing 6% fat and acidified water *ad libitum*. All animal procedures followed Association for Assessment and Accreditation of Laboratory Animal Care guidelines and were approved by institutional Animal Care and Use Committee. For all experiments described in this paper, wildtype and *Celf4* mutant mice were analyzed on an isogenic 129/SvImJ (129S1) strain background. *Celf4* genotyping was performed as previously described [24].

### Splice-junction microarray

Three biological replicates of wildtype and *Celf4* null brain were collected from postnatal day 6 mice. The high-resolution AltSplice microarrays were produced by Affymetrix (Santa Clara, CA, SA), the cDNA samples were prepared using the GeneChip WT cDNA Synthesis and Amplification kit (Affymetrix 900673), followed by GeneChip Hybridization, Wash, and Stain Kit (Affymetrix 900720) using Affymetrix GeneChip Fluidics Station 450 and scanned on Affymetrix GeneChip Scanner 3000 7G. The resulting cel files were analysed with the version 3 of ASPIRE [73]. For examination of splicing, the fold change in gene expression was determined by comparing the average transcript abundance in the three biological replicates of wildtype and *Celf4* null brain, and the *p*-value was determined using Student's t-test (paired, unequal variance). For examination of transcript-level expression, we fit and tested analysis of variance models using J/Maanova (http://churchill.jax.org/software/jmaanova.shtml).

### iCLIP

Whole brain dissected from two *Celf4* null and three wildtype mice aged 6 weeks was split into left and right hemispheres and then dissociated in PBS, UV-crosslinked and collected by centrifugation. The iCLIP method was done as previously described [41]. Briefly, crosslinked brain tissue was dissociated in lysis buffer, sonicated and subjected to partial RNase I digestion (final dilution 1∶100,000). CELF4 complexes were immunopurified with 0.9 µg anti-CELF4 polyclonal antibody (Sigma HPA037986, St. Louis, MO) conjugated to 100 µl protein A Dynabeads (Invitrogen, Carlsbad, CA, USA). While being immobilized on the beads, RNAs bound to CELF4 were dephosphorylated at their 3′ end followed by ligation of the DNA linker 5′-rAppAGATCGGAAGAGCGGTTCAG/ddC/-3′. After 5′ end radiolabeling, crosslinked CELF4-RNA complexes were size-separated by SDS-PAGE and transferred onto nitrocellulose membrane. The regions corresponding to 60–200 kDa on the autoradiogram were excised from the nitrocellulose and bound RNAs were released by proteinase K treatment. RNAs were reverse-transcribed (File S1) and cDNAs were size-selected from a 6% TBE-urea gel (Invitrogen). Purified cDNAs were circularized, linearized by restriction digestion and PCR amplified.

### High-throughput sequencing and analysis of iCLIP data

High-throughput sequencing of iCLIP cDNA libraries was performed on an Illumina GA-IIx (run length 54 nt). The iCLIP libraries contained a 4-nt experimental barcodes plus a 5-nt random barcode, which allowed multiplexing and the removal of PCR duplicates, respectively (File S1). All genomic analyses were performed using the mouse genome version mm9/NCBI37 with annotations taken from Ensembl (version 60). The iCLIP data were mapped using Bowtie [74] and randomers were evaluated as described previously [73]. For assessing the genomic distribution of iCLIP crosslink nucleotides, we used the following hierarchy: ncRNA>3′ UTR>5′ UTR>exon>intron>antisense>intergenic, as defined by Ensembl 59 (Figure 1C). Identification of significantly clustered crosslink sites was described earlier [75]. After defining the clusters, we used the cDNA count at each crosslink site in the cluster to identify the position that represents the centre of mass. We then extended this position by 15 nts on each side to generate 31 nucleotide windows, which were used to determine the occupancy at each crosslink cluster. Sum of cDNA counts in each window was divided by the total DNA count of the iCLIP library. Occupancy in windows was then determined by further dividing by the RNAseq FPKM value of the gene containing the window. FPKM was calculated by mapping ERR033018 and ERR033019 datasets [ENA:ERP000614] using TopHat in a way where each read from the paired-end sequencing was mapped separately, and the FPKM was then calculated from pooled data using Cufflinks. iCLIP binding motif analysis was done as described previously [75], except that the range of binding positions was extended to 10 nt and 30 nt in each direction. The z-score was calculated for each pentamer as: (occurrence in iCLIP sequences – average occurrence in randomized control sequences)/standard deviation of occurrence in randomized control sequences. DREME software [42] was then used identify the motif that was most highly enriched in wildtype brain compared with *Celf4* null brain.

### Analysis of polysome distribution and mRNA localization

For material from polysome fractions and dissected hippocampus, The Jackson Laboratory Gene Expression Service prepared mRNA sequencing libraries using the Illumina TruSeq methodology. RNA was extracted using TRIzol (Invitrogen, CA). For mRNA-Seq, mRNA was purified from total RNA using biotin tagged poly dT oligonucleotides and streptavidin coated magnetic beads followed by QC using an Agilent Technologies 2100 Bioanalyzer (Agilent Technologies, Santa Clara, CA, USA). The mRNA was then fragmented and double stranded cDNA was generated by random priming. The ends of the fragmented DNA were converted into phosphorylated blunt ends. An ‘A’ base was added to the 3′ ends. Illumina-specific adaptors were ligated to the DNA fragments. Using magnetic bead technology, the ligated fragments were size selected and then a final PCR was performed to enrich the adapter-modified DNA fragments since only the DNA fragments with adaptors at both ends will amplify. The sequencing library was first validated using an Agilent Technologies 2100 Bioanalyzer to characterize DNA fragment sizes and concentration. The concentration of DNA fragments with the correct adapters on both sides was then determined using a quantitative PCR strategy, following the kit manufacturer's protocol (Kapa Biosystem, Cambridge, MA). Following library quantitation, libraries were diluted and pooled as necessary. Using the Illumina cBot, libraries were added to the flow cells and clusters were generated prior to 100 bp paired end sequencing on the Illumina HiSeq 2000 (Illumina, San Diego, CA, USA). During and after the sequencing run, sequence quality was monitored using the real time analysis (RTA) and sequence analysis viewer (SAV) softwares available by Illumina. Following sequencing, demultiplexed fastQ files were generated using the Illumina CASAVA software.

FastQ files were aligned to the C57BL/6J reference genome on a high performance computing cluster using Tophat (http://tophat.cbcb.umd.edu/) for the alignment and Cufflinks (http://cufflinks.cbcb.umd.edu/) for isoform assembly and quantitation, except that frequency of reads per kilobase was normalized based on quartile instead of the total number of mapped reads. Differential expression analysis for microarray data was done using J/Maanova (http://churchill.jax.org/software/jmaanova.shtml) and for RNAseq using the R stats package (www.R-project.org), including linear models, ANOVA and permutation shuffling. Further analysis was done using JMP (SAS Institute, Inc.) or Microsoft Excel (Microsoft Corp). The analysis workflows for RNAseq differential expression analyses and sample R-scripts are summarized in File S10.

### Hippocampal CA1 dissection

Four-week old mice were euthanized by cervical dislocation. Brains were quickly removed and transferred into ice-cold solution containing: 210 mM sucrose, 3 mM KCl, 1 mM CaCl_2_, 3 mM MgSO_4_, 1 mM NaH2PO4, 26 mM NaHCO3, 10 mM glucose, saturated with 95% O_2_ and 5% CO_2_. Coronal slices were cut at 300 µm on a vibrating microtome (VT 1200, Leica Microsystems, Germany). A patch pipette was used to dissect either cell bodies from CA1 stratum pyramidale or neuropil from CA1 stratum radiatum under a dissecting microscope (illustrated in Figure S2). Tissue was placed directly in TRIzol reagent (Invitrogen) and RNA was prepared as directed.

### Polysome preparations

Four-week old mice were euthanized by cervical dislocation, the brain was rapidly removed from the skull and placed in 1.5 ml of ice-cold lysis buffer (20 mM Tris-HCl, pH 7.4, 3 mM MgCl_2_, 10 mM NaCl, 2% sucrose, 0.3% Triton X-100, 2 mM vanadyl ribonucleoside complexes [VRC] supplemented with protease inhibitors (complete mini, EDTA-free, Roche, Indianapolis, IN). From this point forward the material was kept on ice. The brains were homogenized in lysis buffer with 10 strokes of a mechanical dounce homogenizer. The homogenate was then centrifuged at 10,000 *g* for 10 minutes at 4°C, and the supernatant was removed to a fresh tube. The salt concentration of the supernatant was adjusted to 170 mM NaCl and 13 mM MgCl_2_. Where indicated, the lysate was treated with either 30 mM EDTA or 0.1 mg/ml RNase A for 30 minutes on ice. For EDTA treatment, VRC was not included in the lysis buffer. Lysates were then carefully layered onto 15–55% linear density gradients of sucrose in 25 mM Tris-HCl, pH 7.4, 25 mM NaCl, 5 mM MgCl_2_ and 30 mM EDTA where indicated. Gradients were ultracentrifuged in a Beckman Instruments (Fullerton, CA) SW40Ti rotor (Beckman Instruments, Fullerton, CA, USA) at 36,000 rpm for 2 hours and 25 minutes at 4°C. Fractions were collected in 1 ml increments with continuous monitoring at 254 nm using an ISCO detector. For RNA extraction, fractions were collected into 3 ml of 7.7 M guanidine-HCl, 4 ml of absolute ethanol was added, and samples were incubated overnight at −20°C. Samples were centrifuged at 4000 rpm in an Avanti JS5.3 rotor (Beckman Instruments, Fullerton, CA, USA) for 50 minutes at 4°C. The supernatant was removed and 400 µL DEPC-treated H_2_O was added to the pellet. The pellet was resuspended and moved to a microcentrifuge tube. To precipitate the RNA, 1 ml of absolute ethanol, 40 µL of 3 M sodium acetate, pH 5.2, and 20 µg glycogen were added, and the samples were incubated overnight at −20°C. The samples were then centrifuged at 14,000 *g* for 30 minutes at 4°C. Pellets were washed with ice-cold 75% ethanol and air dried for 15 minutes at room temperature. RNA was resuspended in 30 µL DEPC-treated H_2_O. For western blot analysis, the protein in each fraction was TCA-precipitated.

### Antibodies

The following antibodies were used for immunoblotting and immunostaining, anti-Actin (1∶1,000, Abcam ab3280), anti-Camk2a (1∶400; Millipore NB12), anti-Celf4 (1∶200 for immunoblot, 1∶400 for immunostaining; Santa Cruz sc-84712), anti-Map2 (1∶10,000, Abcam ab5392), anti-Stxbp1 (1∶100; Abcam ab3451), anti-Nnat (1∶100; Abcam ab27266), anti-Synj1 (1∶100; Abcam ab19904).

### Immunoblotting

For immunoblots of proteins isolated from polysome fractionation, proteins were separated by SDS-PAGE and transferred to nitrocellulose. Blots were blocked with 5% nonfat milk in TBST (TBS, 0.1% Tween-20), probed with anti-CELF4 antibody in 3% BSA/TBST overnight at 4°C, washed, and incubated with goat anti-rabbit peroxidase-conjugated secondary antibody (1∶15,000, Bio-Rad,; Hercules, CA, USA). Signal was detected by chemiluminescence with the ECL-prime kit (GE Healthcare). The blot was stripped with Restore stripping buffer (Thermo Scientific, Rockford, IL, USA) and reprobed with anti-S6 antibody in 5% milk/TBST overnight at 4°C, washed, incubated with goat anti-mouse peroxidase-conjugated secondary antibody (1∶15,000, Thermo Scientific, Rockford, IL, USA), and signal was detected with the ECL-prime kit (GE Healthcare).

For quantitative immunoblotting, cortical tissue or hippocampal tissue from wildtype and *Celf4* null homozygous mice was homogenized in RIPA lysis buffer (150 mM NaCl, 50 mM Tris-HCl [pH 8], 1% NP-40, 0.5% sodium deoxycholate, 0.1% SDS) supplemented with protease inhibitors (complete mini, Roche) and phosphatase inhibitors (PhosSTOP, Roche) and incubated for 30 minutes at 4°C. The lysate was centrifuged at 4,500 g for 5 minutes at 4°C. Protein in the supernatant was quantified using the Direct Detect system (EMD Millipore). Proteins were separated by SDS-PAGE and transferred to nitrocellulose membranes. Membranes were blocked in 5% milk/TBST for one hour at room temperature then probed with primary antibodies diluted in 3% BSA/TBST for two hours at room temperature or overnight at 4°C. Membranes were then washed three times for five minutes each in TBST followed by incubation with horseradish peroxidase-conjugated secondary antibodies (goat anti-mouse or goat anti-rabbit, Bio-Rad) diluted in 5% milk/TBST for one hour at room temperature. Signal was detected with the ECL prime kit (GE Healthcare, Piscataway, NJ, USA) using the G:BOX Chemi XT4 system equipped with a Synoptics 4.2 MP cooled CCD camera (Syngene, Frederick, MD, USA). Accurate band sizes were ascertained by also visualizing ProSieve Color protein markers that had been present in the gel and transferred with the sample proteins (Lonza, Basel, Switzerland). Before reprobing, the blot was incubated in stripping buffer (62.5 mM Tris-HCl, 100 mM β-mercaptoethanol, 2% SDS, pH 6.7) at 50°C for 30 minutes, followed by two washes for ten minutes each in TBST. As a loading control, blots were probed with anti-actin antibody. For quantitation, images collected from the G:BOX were imported into ImageJ and optical densities (ODs) of the bands were measured. The OD of each sample was normalized to the OD of the corresponding actin band.

### Immunostaining

Cryosections: Adult mice were anesthetized with a lethal dose of tribromoethanol and perfused with phosphate-buffered saline (PBS pH 7.4). Brains were removed from the skull, cut coronally, and frozen in O.C.T. (Tissue-–Tek, Torrance, CA). Then, 15 µm thick sections from the cortex were collected onto lysine-coated Colorfrost Plus slides (Fisher Scientific, Pittsburgh, PA, USA) and stored at −80°C. Slides were thawed and dried for 30 minutes at room temperature. Tissue sections were re-hydrated in PBS, fixed 10 minutes in 1% paraformaldehyde in PBS, rinsed three times in PBS, blocked in 0.3% Triton-X-100, 1% bovine serum albumin (BSA) and 10% normal goat serum (NGS) in PBS for 2 hours at room temperature, and incubated in primary antibodies diluted in 0.3% Triton-X-100, 1% BSA and 3% NGS in PBS for 2 days at 4°C. Sections were washed three times for 5 minutes each in PBST (PBS, 0.05% Tween-20) and incubated for 2 hours at room temperature in secondary antibody. The sections were washed as before, followed by a wash in PBS and incubated in DAPI diluted in PBS for 5 minutes. The sections were mounted in Prolong Gold antifade reagent (P36930; Invitrogen). The following secondary antibodies were used to visualize the immunoreactions: AlexaFluor 488-conjugated goat anti-mouse (1∶1000; Invitrogen A11017) and AlexaFluor 555-conjugated goat anti-rabbit (1∶1000; Invitrogen A21430).

Free-floating sections: Adult mice were deeply anesthetized and perfused with PBS, followed by 4% paraformaldehyde in PBS. Brains were removed from the skull, post-fixed overnight in the same fixative (4°C), rinsed in PBS, cut sagitally and sectioned at 50 µm on a vibrating blade microtome. When antigen retrieval was necessary the tissue sections were first treated with 0.2 mg/ml pepsin (Sigma P6887) in 0.2 M HCl at 37°C for 10 minutes and washed 3 times in PBS at room temperature. Then sections were incubated in a blocking buffer of 0.3% Triton-X-100, 1% BSA and 10% NGS in PBS for 2 hours at room temperature and transferred into primary antibody diluted in blocking buffer with 3% NGS. After incubating for 2 days at 4°C, sections were washed three times for 10 minutes each in PBST (PBS, 0.05% Tween 20) and incubated for 2 hours at room temperature in secondary antibody diluted in blocking buffer with 3% NGS. The sections were washed as before, followed by a wash in PBS and incubated in DAPI diluted in PBS for 5 minutes. The sections were mounted onto lysine-coated Colorfrost Plus slides (Fisher Scientific) in Prolong Gold antifade reagent (Invitrogen).

Images were collected using a Leica SP5 confocal microscope equipped with a 63x PlanApo objective (N.A. 1.4; Leica Microsystems, Germany). The same laser and detection parameters were used for wild type and mutant sections. To quantify immunostaining of CELF4 targets, images were imported into ImageJ. Z-stacks comprising the immunostaining signal were summed into a single projection (the same number of z-stacks were summed for wildtype and mutant). Cell bodies or neuropil (N = 10–20) were manually selected and fluorescence intensity was measured. For each image, background selections (N = 10) were measured, and the average background for the image was subtracted from the cell measurements. Images were not processed in any way prior to quantification. For figure presentation, brightness and contrast were minimally adjusted and equivalent adjustments were made to both wildtype and mutant images.

### Quantitative RT–PCR (qPCR)

Total RNA was prepared from the cortex and hippocampus of adult mice between 8–9 weeks of age using TRIzol reagent (Invitrogen) and treated with DNase I (Promega) according to the manufacturers' suggested conditions. Two micrograms of RNA was transcribed with avian myeloblastosis virus reverse transcriptase. The cDNA from three homozygous mutants and three wildtype littermates was diluted 20-fold and 1.5 µl was added to 13.5 µl of Sybr Green PCR mix (Finnzymes DyNAmo HS SYBR Green qPCR Kit, New England Biolabs) with primers (File S9). The PCR amplifications were run in triplicate and monitored by an ABI Prism 7000 sequence detector (Applied Biosystems, Foster City, CA). The correct PCR amplification was confirmed by the dissociation curve function of the ABI machine and by agarose gel electrophoresis. Relative expression was calculated as fold change, or 2^ΔΔCt^, where ΔΔCt is the difference in cycle number to threshold (Ct, averaged from triplicates) between experimental and reference (actin) amplicons.

### Primary neuronal culture

Hippocampi from embryonic day 16 (E16) mice were dissected under a dissecting microscope in cold artificial cerebrospinal fluid (ACSF, 119 mM NaCl, 5 mM KCl, 1 mM MgCl_2_, 30 mM dextrose, 25 mM HEPES, pH 7.4, without calcium). Following dissociation with papain in ACSF at 37°C for 15 min, 2×10^5^ hippocampal neurons were seeded on 12 mm poly-lysine treated coverslips (size #1.5). The plating medium was Neurobasal medium containing containing 50 U/ml penicillin, 50 g/ml streptomycin, and 2 mM GlutaMAX, supplemented with 2% SM1 (Stemcell Technologies) and 5% heat-inactivated horse serum (Invitrogen). Twenty-four hours after initial plating, the original plating medium was replaced with fresh plating medium. Neurons were then fed twice weekly with Neurobasal media (see above) supplemented with 2% SM1 starting at 4 days *in vitro* (DIV4).

### Immunocytochemistry

Neurons at DIV14 were washed in warm ACSF and fixed in ice-cold 4% paraformaldehyde with 4% sucrose in PBS for 15 minutes at 4°C. Fixation was quenched in 0.1 M glycine in PBS for 5 minutes at room temperature, and cells were washed two times for 5 minutes in PBS at room temperature. Neurons were permeabilized in 0.25% Triton X-100 in PBS for 5 minutes at room temperature, blocked (10% normal goat serum, 0.1% Triton X-100, in PBS), then incubated with anti-CELF4 and anti-MAP2 primary antibodies overnight at 4°C. After three washes in PBS for five minutes each at room temperature, secondary antibodies (AlexaFluor 488-conjugated goat anti-rabbit and AlexaFluor 555-conjugated goat anti-chicken, Invitrogen) diluted 1∶500 in PBS were added for one hour at room temperature. Neurons were then washed and stained with DAPI (1∶1500, Sigma) and mounted in ProLong Gold (Invitrogen). Images were collected using a Leica SP5 confocal microscope equipped with a 63x PlanApo oil immersion objective (N.A. 1.4, Leica Microsystems, Germany).

### Data access

Accession number for the CELF4 iCLIP data in ArrayExpress: (E-MTAB-1162). Accession number for the microarray data in ArrayExpress: (E-MTAB-1163). Accession number for the polysome data in NCBI Bioproject: PRJNA168524. Accession number for the hippocampal dissection data in NCBI Bioproject: PRJNA168525.

## Supporting Information

Figure S1 CELF4 is not present in synaptosomes. Synaptosomes were isolated from wildtype mouse cortical brain homogenates. Immunoblot using antibody against CELF4 shows robust signal in input (25 µg total protein), a very faint band in membranous material, and no band in the synaptosome fraction.
(DOCX)

Figure S2 Illustration of dissection of CA1-hippocampus used for RNAseq experiment. As described in the text.
(DOCX)

File S1 iCLIP primers and summaries, qPCR primers. Text file comprised of five small tables showing primers used for iCLIP and qPCR validation studies, as well as summary statistics for iCLIP results.
(DOCX)

File S2 Normalized RNAseqdata & iCLIP occupancy & ranks. Spreadsheet that shows the RNA sequencing data from the sucrose gradient fractionation and hippocampal dissection experiments, normalized as described in the text, as well as the CELF4 iCLIP occupancy values and relative ranks from the iCLIP-seq experiments. Genes are listed by Ensembl Gene ID.
(XLSX)

File S3 GO annotation clustering for top vs. bottom 500 targets ranks. Workbook with multiple worksheets that summarizes the first pass at functional annotation clustering of CELF4 iCLIP targets, comparing the top 500 ranking targets with the bottom 500 ranking targets. Genes are listed by the Mouse Genome Database ID as well as by their International Nomenclature gene symbols.
(XLSX)

File S4 CELF4 microarray transcript level normalized data and fstats, and qPCR data and summary statistics. Workbook with multiple worksheets that shows normalized microarray data and results of MAANOVA (F statistics, p-values) and the qPCR validation data. Genes are listed by Ensembl Gene ID.
(XLSX)

File S5 Fstats & p values for interaction anova - whole set. Workbook with multiple worksheets that shows F-statistics and p-values for the interaction ANOVA analysis of subcellular fractionation or dissection RNAseq experiments. These data are for the whole transcriptome set. Genes are listed by Ensembl Gene ID.
(XLSX)

File S6 Fstats & p values for interaction anova - CA1 only set. Workbook with multiple worksheets that shows F-statistics and p-values for the interaction ANOVA analysis of subcellular fractionation or dissection RNAseq experiments. These data are for the subset of the whole transcriptome filtered for CA1 synaptic transcripts, as described in the text. Genes are listed by Ensembl Gene ID.
(XLSX)

File S7 GO annotation clustering of interaction models. Workbook with multiple worksheets that shows functional annotation clustering of the highest 2000 ranking CELF4 iCLIP targets from the *Celf4* genotype-dependent interaction ANOVA analyses of subcellular fractionation or dissection RNAseq experiments, comparing the top 50% F-statistics vs. bottom 50% F-statistics for GO categories. Genes are listed by the Mouse Genome Database ID as well as by their International Nomenclature gene symbols.
(XLSX)

File S8 Posthoc analyses of 142 cb vs. np enriched genes from GO models. Workbook with multiple worksheets that shows the results of the GO analysis of *Celf4*-genotype dependent subcellular gene expression shifts of CELF4 targets. Genes are listed by the Mouse Genome Database ID.
(XLSX)

File S9 Synaptosome methods. Text file that describes the methods for synaptosome analysis, shown in Figure S1.
(DOC)

File S10 RNAseq workflows and R-scripts. A pdf file that shows an overview of the workflow used for analyzing RNAseq data in the subcellular fractionation experiments, as well as R-script examples used for ANOVA and permutation analyses.
(PDF)
