# Supplementary material for: CELF4 Regulates Translation and Local Abundance of a Vast Set of mRNAs, Including Genes Associated with Regulation of Synaptic Function
Source: PLoS Genet. 2012 Nov 29;8(11):e1003067. doi: 10.1371/journal.pgen.1003067 (PMC3510034; doi:10.1371/journal.pgen.1003067)
Supplement: Figure S1 — CELF4 is not present in synaptosomes. Synaptosomes were isolated from wildtype mouse cortical brain homogenates. Immunoblot using antibody against CELF4 shows robust signal in input (25 µg total protein), a very faint band in membranous material, and no band in the synaptosome fraction. (DOCX) [file pgen.1003067.s001.docx]

**Figure S1**


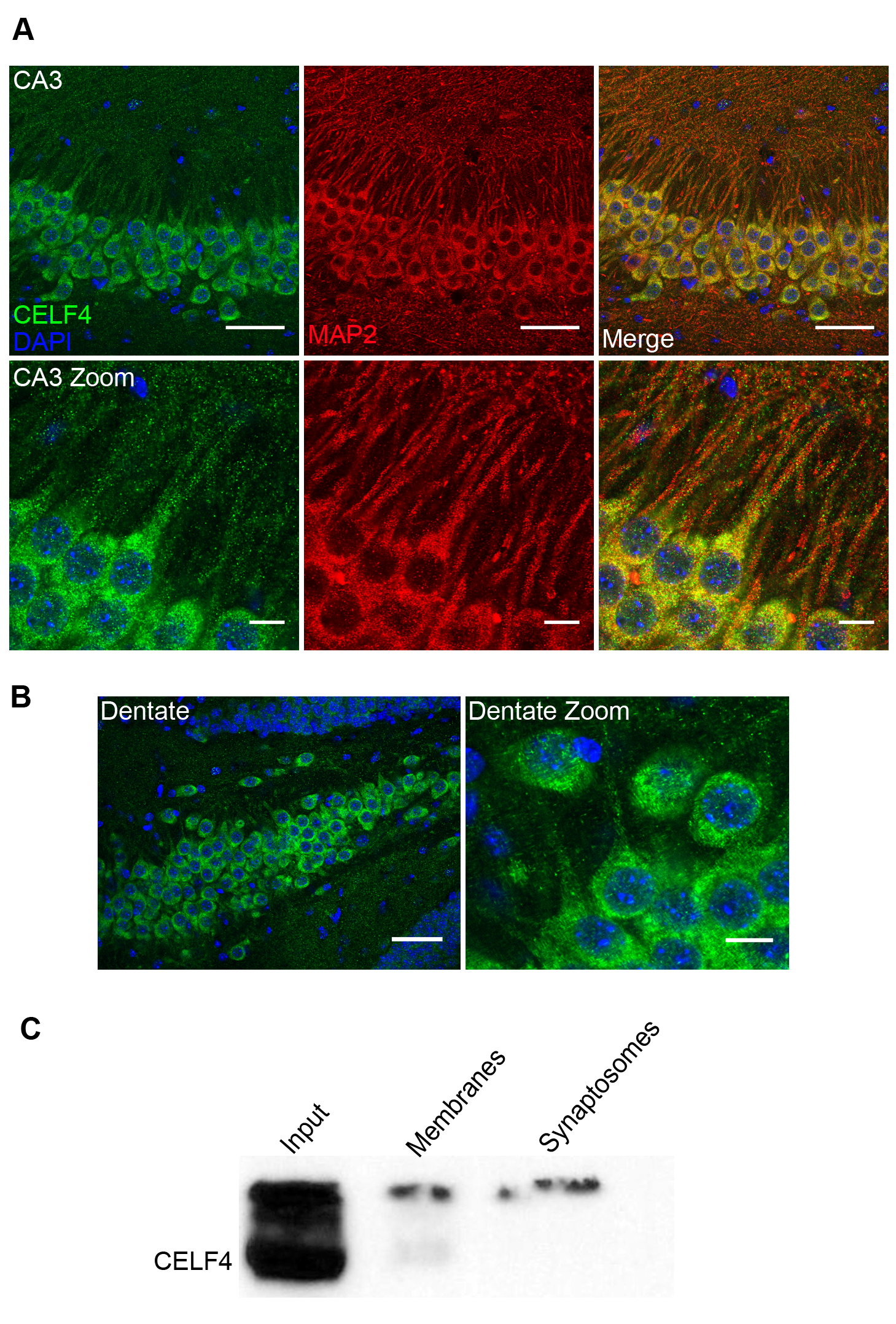


Figure S1. CELF4 is not present in synaptosomes

Synaptosomes were isolated from wildtype mouse cortical brain homogenates. Immunoblot using antibody against CELF4 shows robust signal in input (25 μg total protein), a very faint band in membranous material, and no band in the synaptosome fraction.
