# Supplementary material for: CELF4 Regulates Translation and Local Abundance of a Vast Set of mRNAs, Including Genes Associated with Regulation of Synaptic Function
Source: PLoS Genet. 2012 Nov 29;8(11):e1003067. doi: 10.1371/journal.pgen.1003067 (PMC3510034; doi:10.1371/journal.pgen.1003067)
Supplement: Figure S2 — Illustration of dissection of CA1-hippocampus used for RNAseq experiment. As described in the text. (DOCX) [file pgen.1003067.s002.docx]

**Figure S2**

Illustration of dissection of CA1-hippocampus used for RNAseq experiment, as described in the text.
