# Supplementary material for: CELF4 Regulates Translation and Local Abundance of a Vast Set of mRNAs, Including Genes Associated with Regulation of Synaptic Function
Source: PLoS Genet. 2012 Nov 29;8(11):e1003067. doi: 10.1371/journal.pgen.1003067 (PMC3510034; doi:10.1371/journal.pgen.1003067)
Supplement: File S1 — iCLIP primers and summaries, qPCR primers. Text file comprised of five small tables showing primers used for iCLIP and qPCR validation studies, as well as summary statistics for iCLIP results. (DOCX) [file pgen.1003067.s003.docx]

**Table S1. Reverse transcription primer sequences.**

| **Condition_**  **BrainID_**  **hemisphere** | **Primer** |
| --- | --- |
| KO_11739_R (null1) | 5'-X33NNAACCNNNAGATCGGAAGAGCGTCGTGgatcCTGAACCGC-3' |
| KO_11743_R (null2) | 5'-X33NNCGCCNNNAGATCGGAAGAGCGTCGTGgatcCTGAACCGC-3' |
| KO_11739_L (null3) | 5'-X33NNACAANNNAGATCGGAAGAGCGTCGTGgatcCTGAACCGC-3' |
| KO_11743_L (null4) | 5'-X33NNGCCANNNAGATCGGAAGAGCGTCGTGgatcCTGAACCGC-3' |
| WT_11740_R (wt1) | 5'-X33NNACAANNNAGATCGGAAGAGCGTCGTGgatcCTGAACCGC-3' |
| WT_11745_R (wt2) | 5'-X33NNGCCANNNAGATCGGAAGAGCGTCGTGgatcCTGAACCGC-3' |
| WT_11742_L (wt3) | 5'-X33NNAACCNNNAGATCGGAAGAGCGTCGTGgatcCTGAACCGC-3' |
| WT_11745_L (wt4) | 5'-X33NNCGCCNNNAGATCGGAAGAGCGTCGTGgatcCTGAACCGC-3' |

**Table S2. Number of Celf4 iCLIP sequence reads mapping to the genome and, subsequently, to the transcriptome.**

| **Condition_**  **BrainID_**  **hemisphere** | **# Reads matched**  **with barcode** | **# Reads mapped as single hits to the genome (%)** |
| --- | --- | --- |
| KO_11739_R (null1) | 3324526 | 2338040 (70.3) |
| KO_11743_R (null2) | 1484957 | 1079053 (72.7) |
| KO_11739_L (null3) | 1132397 | 762076 (67.3) |
| KO_11743_L (null4) | 1542791 | 1149303 (74.5) |
| WT_11740_R (wt1) | 5118837 | 4018236 (78.5) |
| WT_11745_R (wt2) | 6419078 | 5053357 (78.7) |
| WT_11742_L (wt3) | 3567708 | 2804200 (78.6) |
| WT_11745_L (wt4) | 5018717 | 3749411 (74.7) |

**Table S3. Correlation of unique clusters between iCLIP replicates**

|  | null1 | null2 | null3 | null4 | wt1 | wt2 | wt3 | wt4 |
| --- | --- | --- | --- | --- | --- | --- | --- | --- |
| null1 | 1.0000 | 0.9537 | 0.9409 | 0.9548 | 0.9035 | 0.8862 | 0.8829 | 0.8875 |
| null2 | 0.9537 | 1.0000 | 0.9345 | 0.9447 | 0.8955 | 0.8799 | 0.8776 | 0.8803 |
| null3 | 0.9409 | 0.9345 | 1.0000 | 0.9363 | 0.8792 | 0.8609 | 0.8623 | 0.8624 |
| null4 | 0.9548 | 0.9447 | 0.9363 | 1.0000 | 0.8947 | 0.8772 | 0.8770 | 0.8793 |
| wt1 | 0.9035 | 0.8955 | 0.8792 | 0.8947 | 1.0000 | 0.9806 | 0.9762 | 0.9787 |
| wt2 | 0.8862 | 0.8799 | 0.8609 | 0.8772 | 0.9806 | 1.0000 | 0.9772 | 0.9830 |
| wt3 | 0.8829 | 0.8776 | 0.8623 | 0.8770 | 0.9762 | 0.9772 | 1.0000 | 0.9779 |
| wt4 | 0.8875 | 0.8803 | 0.8624 | 0.8793 | 0.9787 | 0.9830 | 0.9779 | 1.0000 |

**Table S4. Pentamer motif analysis of *Celf4* null iCLIP tags compared with wt**

| pentamer | z-score (wt) | z-score (null) |
| --- | --- | --- |
| TGTGT | 616.647 | 380.885 |
| TGTTT | 504.892 | 281.01 |
| GTGTG | 465.641 | 309.467 |
| TTTGT | 457.409 | 244.025 |
| TTGTT | 433.145 | 199.973 |
| TATGT | 406.931 | 226.767 |
| TGTAT | 367.358 | 193.47 |

**Table S5. Primer sequences used for Quantitative real-time reverse transcription PCR**

ActinF, 5′-CATTGCTGACAGGATGCAGAA-3′; ActinR, 5′-GCCACCGATCCACACAGAGT-3′

BsnF, 5’-CTATCCCAGGCCTGAGCCAAA-3’; BsnR, 5’-TTGGCTTCTGCCTCTCCACAGAT-3’

Calm1F, 5’-AAGGCTGTCACCAAATCCCACA-3’; Calm1R, 5’-ATGGTGTGCTCAAGTCCACAGA-3’

Cdk5r1F, 5’-GAGTCATGAAGCTCAGTTTGGC-3’; Cdk5r1R, 5’-TGACGGATGGTCAGTGCATTT-3’

Cplx1F, 5’-ATGGCCCTCTCTTGAGTCAATCCT-3’; Cplx1R, 5’-ACGAGGACAGACAGACAGGTAACA-3’

Cplx2F, 5’-ACCTCTTTCCCACAACTGTGCT-3’; Cplx2R, 5’-TTTGGTCAGGTTCAAGGGAGTTCG-3’

Htr2cF, 5’-GCTGCTCTCTAAGAATTAAGTACC-3’; Htr2cR, 5′-GGGCATAAGAACTAGCATCATG -3’

ImpactF, 5’- AGGTTAACTTCCCAGCCACTGT-3’; ImpactR, 5’- TGCTGGTGGAACCATGATGAAC-3’

Kcnd2F, 5’-TTCTGTGCACTTACAATGAGCTG-3’; Kcnd2R, 5’-CCCATGAGAAACACTGTGGTG-3’

Mtap2F, 5’-ATCTGTGTCTCCTGCACCCTTT-3’; Mtap2R, 5’-GGCACTAAGACAGGGTTACAGCTT-3’

NapbF, 5’-AACTGTGGAGAATCTGGCAGCA-3’; NapbR, 5’-TCCCTTGCCTGAGAGCAACTTA-3’

NdnF, 5’-ACAAAGAAGGCCCTGGAGAGTT-3’; NdnR, 5’-TGCTTCCTGTGCCAGTTGAAGT-3’

NnatF, 5’-TCACGCAGCAGTTGTGGTCAAT-3’; NnatR, 5’-TCCCTGTCTCCAGGAGCTTACAAT-3’

NsfF, 5’-CTCTCTCCACACACTCGCTCTG-3’; NsfR, 5’-TTGACCTTCGGGCAGGTGGTAG-3’

Rab3cF, 5’-TCTCTGCGTGCTTGAGAGCAAT-3’; Rab3cR, 5’-TTCCAGAGAGCCATGGTGGTGTTT-3’

Scamp1F, 5’-GCTCGGTTCATTGCTCTAGGGAT-3’; Scamp1R, 5’-AACATGCAACATGAGAGGGCCA-3’

Scn2a1F, 5’-AACTGCCACACCTGCCATATTT-3’; Scn2a1R, 5’-CCCAAACGTAAACCCAGTTACA-3’

Slc1a2F, 5’-GACAAGAACCACAGGGAAAGCA-3’; Slc1a2R, 5’-TTTCCATTGGCCGCCAGAGTTA-3’

Slc12a5F, 5’-TAGTGTTTCGGTATTTCGCATCCC-3’; Slc12a5R, 5’-AGGAGAGAACTGACCCTCTCCTTT-3’

Snap25F, 5’-CAGTGGCGTTTGCTGAATGACA-3’; Snap25R, 5’-TTGTGGCAGTAGCTCTGTGGAA-3’

Snap91F, 5’-GCTGTTCTGTGGTGAGCCATTT-3’; Snap91R, 5’-TGGGAGGACTTGAGTTTGTCCA-3’

SncaF, 5’-AAAGTCTTCCATCAGCAGTGATC-3’; SncaR, 5’-GAAATAAGTGGTAGTCACTTAGG-3’

Stxbp1F, 5’-TGCTGAGTTCTTCTCCTGTGCGAT-3’; Stxbp1R, 5’-GCTTGCATGTGAACTTACTGTGGG-3’

Syn1F, 5’-TCCATCCCATTCAATATACTGGA-3’; Syn1R, 5’-CAGTCACAGATAAACACATTGGC-3’

Synj1F, 5’-TCAGGAAGTCTGGTAGCTGCTGAA-3’; Synj1R, 5’-TGACCAGACCTGGCAAATACACAC-3’

Syt1F, 5’-TGCCCTGTGTGCTTAGACACGTA-3’; Syt1R, 5’-TGCAGTACGAGGGAATGGCAGTAT-3’

VapaF, 5’-GGGAAATGGTGCCTCTTACTGT-3’; VapaR, 5’-CAACAAACTGCCTCACAAGGGA-3’

Zcchc12F, 5’-AAAGAGAGGTGGCCTGACCTAT-3’; Zcchc12R, 5’-AAAGCCACCAAGGAGAGAGGTT-3’

Zfp238F, 5’-AGCCCGATGTGTGGTTCTGAT-3’; Zfp238R, 5’-ACTGTTTCCCATGCTACTCCCA-3’
