# Supplementary material for: CELF4 Regulates Translation and Local Abundance of a Vast Set of mRNAs, Including Genes Associated with Regulation of Synaptic Function
Source: PLoS Genet. 2012 Nov 29;8(11):e1003067. doi: 10.1371/journal.pgen.1003067 (PMC3510034; doi:10.1371/journal.pgen.1003067)
Supplement: File S9 — Synaptosome methods. Text file that describes the methods for synaptosome analysis, shown in Figure S1. (DOC) [file pgen.1003067.s011.doc]

**Synaptosome preparation**

Discontinuous Percoll (GE Healthcare) gradients with 3%, 10% and 23% Percoll layers were prepared in gradient buffer (0.32 M sucrose, 1 mM EDTA, 0.25 mM DTT, 5 mM Tris-HCl, pH 7.4). Mice were euthanized by cervical dislocation, and the brains were quickly removed to ice-cold homogenizing buffer (gradient buffer without Percoll). Brains were homogenized with 10 strokes in a mechanical dounce homogenizer. 2.5 ml homogenizing buffer was added to dilute the protein. Homogenates were centrifuged in a JA-20 rotor (Beckman Instruments, Fullerton, CA, USA) at 3600 rpm for 10 minutes at 4°C in 12 ml polycarbonate centrifuge tubes. The supernatant was removed to a fresh tube, and an input sample was taken. 2 ml of the supernatant was layered onto the top of the Percoll gradient. Gradients were centrifuged in a JA-20 rotor at 20,000 rpm for 5 minutes exactly at 4°C with deceleration set to the slowest setting. Fractions containing myelin and membranes were removed from the interface between the 3% and 10% layers and fractions containing synaptosomes were removed from the interface between the 10% and 23% layers. Each fraction was diluted four-fold with ice-cold sucrose-EDTA buffer (0.32 M sucrose, 4 mM EDTA, 20 mM Tris, pH 7.4). Fractions were centrifuged in a JA-20 rotor at 15,000 rpm for 5 minutes at 4°C to remove Percoll. Pellets were resuspended and collected into a microcentrifuge tube and centrifuged at maximum speed for 5 minutes at 4°C. The pellet was then resuspended in 50 μl in protein lysis buffer (20 mM Tris-Hcl, pH 7.5, 0.5 mM EDTA, 100 mM NaCl, 0.5% NP-40) plus protease inhibitors (Roche). Protein was quantitated using the Bradford assay (Bio-Rad). Western blotting was performed as described previously in Materials and Methods.
