# Supplementary material for: CELF4 Regulates Translation and Local Abundance of a Vast Set of mRNAs, Including Genes Associated with Regulation of Synaptic Function
Source: PLoS Genet. 2012 Nov 29;8(11):e1003067. doi: 10.1371/journal.pgen.1003067 (PMC3510034; doi:10.1371/journal.pgen.1003067)
Supplement: File S10 — RNAseq workflows and R-scripts. A pdf file that shows an overview of the workflow used for analyzing RNAseq data in the subcellular fractionation experiments, as well as R-script examples used for ANOVA and permutation analyses. (PDF) [file pgen.1003067.s012.pdf]

Workflows for RNAseq analyses (polysomes or hippocamal/CA1 dissection experiment)

Cufflinks 1.3 output (unfiltered) to normalized, filtered expression data (MS Excel)

→ derive "FPK" from FPKM x #_mapped_reads/1M

→ derive the 75% ile (third quartile) for each sample-array

→ determine which sample has the highest value (set to "1")

→ normalize other samples' expression values to this

→ derive log(2) for each expression value

→ arbitrarily exclude genes with very low expression values in either RNAseq or iCLIP occupancy

→ ~14k gene dataset for further analysis

Prepared normalized, filtered data for expression analysis (JMP)

→ reformat data file to stacked

→ rank transform data by sample type

→ derive normal-quantiles from rank-transformed data

Expression analysis: ko vs. wt using linear models and ANOVA (R on Mac Terminal)

→ Rstats script to get f-statistics and p-values solving for genotype, experiment, genotype x experiment interaction terms for each gene

→ Rstats script ("sample" command on above) to get permuted p-values for f-stats

Compare *Celf4* expression changes to CELF4 iCLIP target binding (R on Mac Terminal)

→ rank-transform gene expression f-stats and iCLIP occupancies (excluding ≤0 occupancy)

→ scatter plot to visualize relationships (JMP)

→ Rstats script linear model for dataset where x = f-stat rank and y = iCLIP rank

→ get f-statistics and permutation p-values

```
#This script is for getting fstatistics and uncorrected pvalues of transformed RNAseq data as X
 against genotype (Celf4 null vs. wt; environment or experiment (sucrose gradient fraction pool ID,
 or subcellular compartment) and the interaction between Celf4 genotype and experiment. This
 particular script is setup to examine polysome vs. monosome fraction pools. Scripts for RNA granules
 vs. mono/polysomes or cell body vs. neuropil are similar)
# this reads in the tab delimited text file called "fracsallsomes.txt" containing the data in
 stacked
testExp = read.delim("fracsallsomes.txt",sep="\t",header=T)
# this sets the results that get written to the designated output file
cat(c("id","fstatgeno","fstatenvir","fstatint","pvalgeno","pvalenvir","pvalint\n"), append=T,
 file="fracsallsomes_logqranksep_out.txt")
#"id" is set as the "gene" and its looking at from one to "n" genes so you don't need to specify how
 many. ensIDs=as.vector(unique(testExp$id))
nGenes = length(ensIDs)
for (g in 1:nGenes){
    oneGene = testExp[testExp$id==ensIDs[g],]
# for this particular input it expects each gene to have 20 entries corresponding to the five
 replicates and both genotypes; 10 for monosomes 10 for polysomes
    if (nrow(oneGene)!=20){
    cat(c("Warning: Gene", ensIDs[g], "does not have 20 values\n"),spe="  ")
    }
# the next set of parameters defines Y (fraction) and X (genotype) variables.
# it then sets up the linear models 1-4, and then defines the anova for the genotype effect, the
 'environmental' effect (i.e. which fraction type) and the interaction between them.  It also defines
 what goes to the output (fstats and p values).  note that permutation p values are used, as
 calculated from a separate script
    gtype = as.numeric(oneGene$genotype)
    environ = as.numeric(oneGene$fraction)
    exp = oneGene$logqranksep
    lm1= lm(exp~gtype)
    lm2= lm(exp~environ)
    lm3= lm(exp~gtype+environ)
    lm4= lm(exp~gtype+environ+gtype*environ)
    anovaModelgeno=anova(lm2,lm3)
    anovaModelenvir=anova(lm1,lm3)
    anovaModelint=anova(lm3,lm4)
    fstatgeno= anovaModelgeno$'F'[2]
    fstatenvir= anovaModelenvir$'F'[2]
    fstatint= anovaModelint$'F'[2]
    pvalgeno = anovaModelgeno$'Pr(>F)'[2]
    pvalenvir = anovaModelenvir$'Pr(>F)'[2]
    pvalint = anovaModelint$'Pr(>F)'[2]
    if (pvalgeno>1){
     cat(c("error : Pval > 1 for ", ensIDs[g],"\n"))
    }
    cat(c(ensIDs[g],fstatgeno,fstatenvir,fstatint,pvalgeno,pvalenvir,pvalint,"\n"), append=T,
 file="fracsallsomes_logqranksep_out.txt")
    #cat(c(fstatgeno,fstatenvir,fstatint,pvalgeno,pvalenvir,pvalint,"\n"), append=T,
 file="fracsallsomes_logqranksep_out.txt")
    }
# read the result as table
stats = read.table("fracsallsomes_logqranksep_out.txt",header=T)
# diagnostics this creates a pdf showing the distribution of f statistics
head(stats)
dim(stats)
pdf("somesgene-env-int-pval-hist.pdf",width=3,height=8)
par(mfrow=c(3,1))
hist(stats$pvalgeno,col="grey")
hist(stats$pvalenvir,col="grey")
hist(stats$pvalint,col="grey")
dev.off()
```

```
# This script is to get permutation p values for experimental fstatistics for RNAseq gene expression
 experiments.  most of this is the same as the script that generates the experimental f statistics
 and p values, except that it uses the "sample" command which randomly tests each model by
 permutation testing for n samples, where n is equal to the number of 'genes' in the dataset
testExp = read.delim("fracsallsomes.txt",sep="\t",header=T)
cat(c("id","fstatgeno","fstatenvir","fstatint","pvalgeno","pvalenvir","pvalint\n"), append=T,
 file="permfracsallsomes_logqranksep_out.txt")
#cat(c("fstatgeno", "fstatenvir","fstatint", "pvalgeno", "pvalenvir","pvalint\n"), sep="\t",
 append=T, file="permfracsallsomes_logqranksep_out.txt")
ensIDs=as.vector(unique(testExp$id))
nGenes = length(ensIDs)
for (g in 1:nGenes){
#for (g in 1:100){
    oneGene = testExp[testExp$id==ensIDs[g],]
    if (nrow(oneGene)!=20){
    cat(c("Warning: Gene", ensIDs[g], "does not have 20 values\n"),spe="  ")
    }
    gtype = as.numeric(oneGene$genotype)
    environ = as.numeric(oneGene$fraction)
    exp = sample(oneGene$logqranksep)
    lm1= lm(exp~gtype)
    lm2= lm(exp~environ)
    lm3= lm(exp~gtype+environ)
    lm4= lm(exp~gtype+environ+gtype*environ)
    anovaModelgeno=anova(lm2,lm3)
    anovaModelenvir=anova(lm1,lm3)
    anovaModelint=anova(lm3,lm4)
    fstatgeno= anovaModelgeno$'F'[2]
    fstatenvir= anovaModelenvir$'F'[2]
    fstatint= anovaModelint$'F'[2]
    pvalgeno = anovaModelgeno$'Pr(>F)'[2]
    pvalenvir = anovaModelenvir$'Pr(>F)'[2]
    pvalint = anovaModelint$'Pr(>F)'[2]
    if (pvalgeno>1){
     cat(c("error : Pval > 1 for ", ensIDs[g],"\n"))
    }
    cat(c(ensIDs[g],fstatgeno,fstatenvir,fstatint,pvalgeno,pvalenvir,pvalint,"\n"), append=T,
 file="permfracsallsomes_logqranksep_out.txt")
    #cat(c(fstatgeno,fstatenvir,fstatint,pvalgeno,pvalenvir,pvalint,"\n"), append=T,
 file="permfracsallsomes_logqranksep_out.txt")
    }
# read the result as table
stats = read.table("permfracsallsomes_logqranksep_out.txt",header=T)
# diagnostics
head(stats)
dim(stats)
pdf("gene-env-int-pval-hist.pdf",width=3,height=8)
par(mfrow=c(3,1))
hist(stats$pvalgeno,col="grey")
hist(stats$pvalenvir,col="grey")
hist(stats$pvalint,col="grey")
dev.off()
```

```
#This script is for determining F-statistics and p-values for correlation between Interaction
 Fstatistics and iCLIP binding ranking
#read in the input file
globTab =read.delim("globmods.txt", header=T)
# the input file ('globmods.txt' in this case) has to be ordered by binding rank, least to best.
 we are starting at 4365 because these lower ranked genes have 'negative' binding i.e. nonsensical
start = 4365
end = nrow(globTab)

#this is for the modeling of 'int_s' which is the column header for the rank-ordered fstatistics
 for interaction between celf4 genotype and polysome vs monosome fstats.
#"binding' is the column header for the iCLIP rank order
obs.bindIntS = cor(globTab$binding[start:end],globTab$int_s[start:end])
y = globTab$binding[start:end]
x = globTab$int_s[start:end]
lmObs = lm(y~x)
fs= summary(lmObs)$fstat[1]
obs.bindIntS = c(obs.bindIntS, fs)

#this is to get the permutations for the fstatistic for int_s.  15000 in all
perm.bindIntS =c()
for (i in c(1:15000)){
   perm.cor= cor(sample(globTab$binding[start:end]),globTab$int_s[start:end])
   y = sample(globTab$binding[start:end])
   x = globTab$int_s[start:end]
   lmPerm = lm(y~x)
   fs= summary(lmPerm)$fstat[1]
   perm.bindIntS = c(perm.bindIntS, fs)
}
#############
#this is for the modeling of 'int_g' which is the column header for the rank-ordered fstatistics
 for the interaction between celf4 genotype and RNAgranule vs monopolysome fstats
obs.bindIntG = cor(globTab$binding[start:end],globTab$int_g[start:end])
y = globTab$binding[start:end]
x = globTab$int_g[start:end]
lmObs = lm(y~x)
fs= summary(lmObs)$fstat[1]
obs.bindIntG = c(obs.bindIntG, fs)

#this is to get the permutations for the fstatistic for int_g.  15000 in all
perm.bindIntG =c()
for (i in c(1:15000)){
   perm.cor= cor(sample(globTab$binding[start:end]),globTab$int_g[start:end])
   y = sample(globTab$binding[start:end])
   x = globTab$int_g[start:end]
   lmPerm = lm(y~x)
   fs= summary(lmPerm)$fstat[1]
   perm.bindIntG = c(perm.bindIntG, fs)
}
#############
#this is for the modeling of 'int_d' which is the column header for the rank-ordered fstatistics
 for the interaction between celf4 genotype and cell body vs neuropil dissection fstats
obs.bindIntD = cor(globTab$binding[start:end],globTab$int_d[start:end])
y = globTab$binding[start:end]
x = globTab$int_d[start:end]
lmObs = lm(y~x)
fs= summary(lmObs)$fstat[1]
obs.bindIntD = c(obs.bindIntD, fs)

#this is to get the permutations for the fstatistic for int_d.  15000 in all
perm.bindIntD =c()
```

```
for (i in c(1:15000)){
   perm.cor= cor(sample(globTab$binding[start:end]),globTab$int_d[start:end])
   y = sample(globTab$binding[start:end])
   x = globTab$int_d[start:end]
   lmPerm = lm(y~x)
   fs= summary(lmPerm)$fstat[1]
   perm.bindIntD = c(perm.bindIntD, fs)
}
#this writes the p value and fstatistic on sequential lines for each model int_s, int_g or int_g x
 rank on sequential lines to output file
obs=c(obs.bindIntS,obs.bindIntG,obs.bindIntD)
write.table(obs,file="globmods_out.txt")

#this writes the 15000 permutations for each fstat to output file
 perms=cbind(perm.bindIntS,perm.bindIntG,perm.bindIntD)
write.table(perms,file="permglobmods_out.txt")
```
